# On the consistency of seismically imaged lower mantle slabs

**DOI:** 10.1038/s41598-017-11039-w

**Published:** 2017-09-08

**Authors:** G. E. Shephard, K. J. Matthews, K. Hosseini, M. Domeier

**Affiliations:** 1 Centre for Earth Evolution and Dynamics (CEED), Department of Geosciences, University of Oslo, Oslo, Norway; 2 Department of Earth Sciences, University of Oxford, South Parks Road, Oxford, OX1 3AN United Kingdom

## Abstract

The geoscience community is increasingly utilizing seismic tomography to interpret mantle heterogeneity and its links to past tectonic and geodynamic processes. To assess the robustness and distribution of positive seismic anomalies, inferred as subducted slabs, we create a set of vote maps for the lower mantle with 14 global P-wave or S-wave tomography models. Based on a depth-dependent threshold metric, an average of 20% of any given tomography model depth is identified as a potential slab. However, upon combining the 14 models, the most consistent positive wavespeed features are identified by an increasing vote count. An overall peak in the most robust anomalies is found between 1000–1400 km depth, followed by a decline to a minimum around 2000 km. While this trend could reflect reduced tomographic resolution in the middle mantle, we show that it may alternatively relate to real changes in the time-dependent subduction flux and/or a mid-lower mantle viscosity increase. An apparent secondary peak in agreement below 2500 km depth may reflect the degree-two lower mantle slow seismic structures. Vote maps illustrate the potential shortcomings of using a limited number or type of tomography models and slab threshold criteria.

## Introduction

By utilizing recordings of seismic waves related to earthquakes, seismic tomography provides critical insights into the internal seismic velocity structure of present-day Earth. It follows that by combining seismically imaged features with knowledge from mantle convection and plate tectonics, a time-dependence can be attributed to the imaged mantle structure. Although there exists an increasing number of individual tomography models that can be utilized for these ends, these models vary in their construction and resolving power (e.g. refs 1–4). Differences between tomography models are manifest in seismic anomalies of variable magnitude, geometry, location, depth, and resolution. A measure of the consistency of identified features across models would therefore greatly aid in the identification and evaluation of prominent features.

The broader geoscience community is increasingly using seismic tomography to interpret mantle heterogeneity. It is thus imperative to evaluate the continuity and robustness of seismic anomalies such that both the posteriori interpretations and a priori models can be scrutinized. Plate kinematic models inherently predict regions of subduction and, as commonly assumed, should correspond to positive velocity perturbations (% δlnVs, % δlnVp) in the mantle. As a time-integrated record of internal heterogeneity, seismic tomography models, and in particular the interpretation of slabs, have been used in the construction of relative plate reconstructions as well as testing and refining absolute reference frames, and estimating slab sinking rates (e.g. refs 5–8).

The recent use of seismically identified slabs to validate, test or refine regional relative plate motions includes slabs located under North America^9^, the Pacific^10^, the Indian Ocean region^11–13^, Australia^14^, Siberia^15^, and Greenland^16^, amongst others. However, these studies often focus on a subset of one, two, or a small selection of available tomography models, and are often restricted to a single class of tomography data (e.g. P- or S-waves). Furthermore, seismic tomography is increasingly serving as both an input into mantle convection models e.g. refs 17 and 18, and output to which the temperature structure as predicted by numerical models can be seismically “filtered” (e.g. ref. 19), or directly compared (e.g. ref. 20).

The aim of this paper is to provide an independent set of maps based on the combination of 14 global tomography models, including both S- and P-wave models (Table 1, Methods), to which the continuity or robustness of a particular positive seismic anomaly can be assessed. It is not a direct comparison between the individual tomography models, and it does not serve to dissuade the use of any particular model, replace the existing suite of models, or constitute a tomography model in itself. Rather this study provides a surface-projected global vote map to which the continuity of a feature might be assessed as based on the use of (1) a contour derived from the mean of the positive velocities (MPV), (2) at a given depth (interpolated to 50 km increments), (3) with the layer-dependent mean removed (LDM), and (4) as otherwise presented in available tomography models, without filtering wavelengths, etc. Due to the non-linear dynamics of subduction, the wavelengths considered, and the significant variability in tomography model construction, this analysis does not comment on the spatial resolution^21^ of each of the individual models presented here, nor does it discern the actual existence of a subducted slab or attribute an age or origin to the feature(s). Nonetheless, these vote maps serve as a guide in identifying common features from tomography.

**Table 1:** Summary of global tomography models used in this study.

Model #	Model name*	Reference	Data type	1-D model *Ref below.	Original no. z layers
**P-wave models**					
1	GAP-P4	49, 55	Body	7	29
2	GyPSuM-P	22	Body, geodynamic observations	3, 4	22
3	HMSL-P06	56	Body (long period)	1	18
4	LLNL_G3Dv3	57	Body	1	41
5	MITP_2011	58	Body	1	64
6	PRI-P05	59	Body	2	58
7	UU-P07	60	Body	1	34
**S-wave models**					
8	GyPSuM-S	22	Body, geodynamic observations	3,4	22
9	HMSL-S06	56	Surface, Body	1	18
10	PRI-S05	59	Body	2	58
11	S40RTS	61	Surface, Body, normal models	3	116
12	SAVANI	62	Surface, Body	3	28
13	SEMUCB-WM1	63	Waveform inversions	5	57
14	TX2011	4	Body	6	100 (22 unique)

#1D model references 1. ak135^64^, 2. iasp91^65^, 3. PREM^66^, 4. TNA/SNA^67^, 5 3D starting model: SEMum2 (800 km or shallower) and SAW24B16 (greater than 800 km)^63^, 6 TX2011_ref reference model^4^, 7 laterally averaged P-wave velocity distribution.

*Sources from individual tomography reference websites, and also the IRIS DMC data products repository^68^.

Additional vote map figures and visualization options can be found at an interactive and open access website (http://submachine.earth.ox.ac.uk). As future tomography models are developed and released by the seismological community, and insights into plate reconstructions and the history of subduction are gained from the tectonics and geodynamics communities, this approach can be further adapted, refined and applied.

## Seismic Tomography

Since the development of seismic tomography, the internal structure of Earth has been vastly sampled and imaged at different wavelengths: from local to global scales, and with a variety of spatial resolutions as a function of latitude, longitude or depth. Global tomography models of compressional (P) or shear (S) wave velocity variations have been obtained from different types of data and modeling techniques. Generally, three types of data are used: body-wave traveltime, surface wave dispersion, and normal mode spectral measurements. In global tomography, teleseismic body-wave travel times are the main source of data in constraining the structure of the lower mantle. Surface waves mainly constrain the upper mantle, and normal modes provide information on very large-scale features within the Earth. For brevity, we herein refer to “S-waves” and “P-waves”, even though we technically refer to models of S- or P-wavespeed anomalies. Both S- and P-wave models have long been used to identify deep Earth features (e.g. refs 4 and 22). While the two wave types agree on long wavelength structures, this correlation breaks down at shorter wavelengths^23^. Furthermore, the two wave types have different sensitivities to lateral structure, and while both are sensitive to changes in temperature, the S-waves may be even more so^23^. Our knowledge of structural detail in the lowermost mantle is lacking compared to shallower levels^24^ and is reflected in a greater uncertainty in the imaging of the lowermost mantle structures.

Subducting slabs are one of the first-order features of the upper and lower mantle, contributing strongly to the organization of convective flow, and may interact with the edges of LLSVPs, possibly instigating plume genesis^25^. In the upper mantle (<660 km), recently subducted slabs are characterized by a strong and short wavelength signal^26^. Slab deformation and stalling may occur within the transition zone between the upper and lower mantle due to phase transitions, however, no known phase transitions have been described for the upper part of the lower mantle, and previously described seismic discontinuities between 700–2000 km have been attributed to a viscosity increase (e.g. ref. 27). In the lower mantle (>660 km), the apparent blurring of slabs can be attributed to the effects of thermal diffusion, buckling, advective thickening, and limitations in seismic imaging^28–30^.

Using a different depth-integrated approach to evaluate the robustness of lower mantle features, r﻿e﻿f﻿. 31 applied cluster analysis to tomographic data from five global S-wave models to identify large scale features; notably, two antipodal Large Low Shear Wave Velocity Provinces regions (LLSVP)^32^ and a smaller “Perm” anomaly, surrounded by a contiguous, faster than average region. Subsequently, ref. 33 expanded this analysis to five S-wave and five P-wave models and utilized a moving depth-integrated data window to allow for some assessment of the depth-dependence of lower mantle structures. Here we follow a strongly depth-dependent approach and aim to focus on the smaller wavelength component of fast seismic anomalies.

Choices in data input as well as techniques involved in the inversion, including parameterization and regularization can also yield mantle velocity structures that are regionally variable. It is therefore desirable to discern a feature that is consistent across several tomography models. Whilst a velocity structure in a single given tomography model may be a result of a parameterization choice and/or belong to the “null space,” it is unlikely to be imaged across a suite of different models^31^. Here we compare fourteen global tomography models, which have variable levels of input data overlap and parameterization choices (Table 1, Methods).

## Plate Reconstructions

Crucial constraints on plate motions come from the ocean basins, where tectonic structures such as fracture zones and abyssal hills, and measurements of magnetic anomalies and hotspot tracks directly record the speed and direction of seafloor spreading, and the timing and nature of plate boundary reorganizations. However, due to the persistent recycling of oceanic lithosphere by subduction, these key constraints for reconstructing plate motions are lost over time. By the Early Cretaceous ~60% of the present-day seafloor record is lost^34^ and alternative datasets for constraining plate kinematic histories must be sought.

Considering that the tomographic visibility of the mantle lies around 200 Ma (e.g. ref. 35), though older timescales of ~250 to 300 million years have been proposed (e.g. ref. 5), slabs identified in the lowermost mantle provide such an alternative constraint for refining plate motions, specifically the post-Triassic subduction record. Indeed, recent work^8^ has confirmed that a significant and time-depth progressive correlation exists between reconstructed subduction zone locations and the occurrence of positive wavespeed velocities in the mantle below, providing a solid foundation for further work on a slab-based reference frame. However, such an analysis is dependent on the resolution of the tomography model used and moreover relies on several qualitative and quantitative assumptions, namely that the identified slabs are representative of the true mantle state, have undergone near vertical sinking, did not stall for a significant amount of time in the transition zone, and that the base plate reconstruction itself and key tie-point events such as orogenesis are known accurately.

Traditional “anchor” slabs such as the Farallon, Mongol-Okhotsk and Aegean Tethyan slabs have been recognized (e.g. ref. 5), and can form the basis of subduction-based absolute reference frames. However, these slabs have recently come under reinterpretation as to their origins and ages (e.g. refs 9 and 36), highlighting the importance of a renewed assessment of slab identification.

## Results

### Depth-dependent variation in mean positive value (MPV)

**Figure 1:**
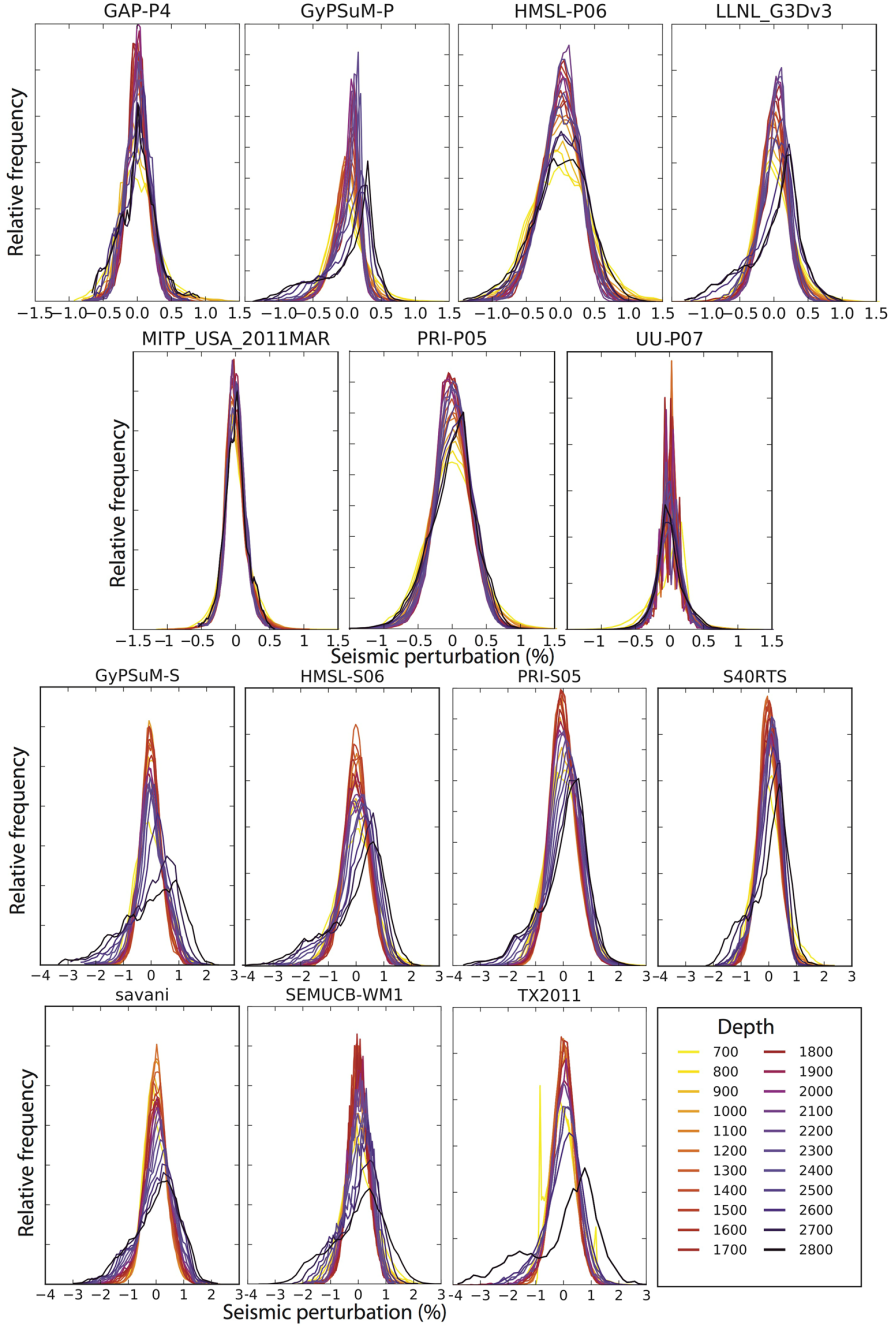
Distribution of velocity anomalies for each tomography model. Histograms for each of the tomography models in 100 km increments, after removal of the LDM.

Figure 1 shows histograms of the seismic velocity anomalies for each of the tomography models at depth, before the positive wavespeeds have been extracted but after the LDM (layer dependent mean; see Methods) removal. Overall, the P-wave models show a smaller range of % wavespeed perturbations (amplitudes) than the S-wave models; i.e. the wavespeed distribution is tighter about 0. Most models exhibit a shift from normally distributed wavespeeds in the shallowest depths, to negatively skewed distributions in the lower mantle (i.e. a lengthened tail in the negative wavespeeds and an increased relative frequency of positive wavespeeds). Furthermore, this effect in the S-wave models is pronounced, leading to a long tail in the negative wavespeed space, in excess of −3% in some models.

**Figure 2:**
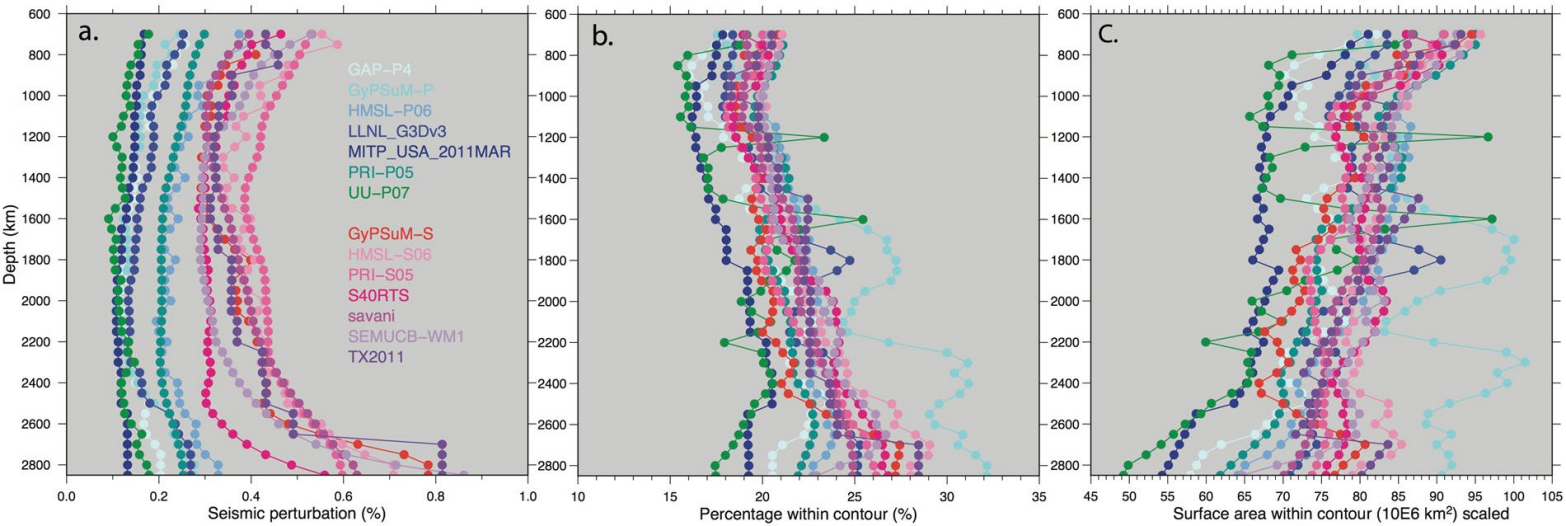
Contouring-related values for each tomography model. (**a**) Mean positive value (MPV) as a function of depth for each of the seismic tomography models discussed in the text. P-wave models grouped in blues and S-wave models in pinks/purples. The reference case grids used here remove the mean for each depth and model (LDM). (**b**) Corresponding percentage of surface area within the MPV contour for each tomography model at each depth i.e. percentage of grid identified as a potential “slab”, not scaled between depths. (**c**) As in (**b**) but scaled to the radius of the Earth.

Figure 2a shows the variability in the depth-dependent mean positive value (MPV) for each tomography model with the LDM removed (Figure S1, Table S2). The depth-dependent MPV profiles range from near-vertical to strongly convex with depth. The P-wave models generally present a lower MPV (i.e. less positive contour value) (average 0.18%) than for the S-wave models (0.41%). The S-wave models also exhibit a larger relative increase in the MPV in the lowermost mantle, below around 2200 km.

Figure 2b shows the corresponding depth-dependent variability in the surface area associated with the wavespeeds that are equal to or exceed the MPV for each tomography model (Table S3). On average for a given tomography model at a given depth, the surface area contoured is 21%, with the lowest coverage at 1000 km depth (18%, *σ 1*.*42*) and the maximum at 2700 km (25%, *σ 3*.*61*). There is not a significant distinction between the proportion of the average area covered by the P-waves combined (21%) versus the S-waves combined (22%), but there is variability between the individual models. Figure 2c shows these same results according to their depth-scaled surface area. Overall, with increasing depth the amount of surface area identified by the MPV is reduced. When viewed in isolation, this panel might suggest that the area of slabs decreases as you descend in the lower mantle, however, by considering the vote maps, an improved insight into the “agreement” of the most robust features can be discussed.

### Vote maps - a visual comparison

#### Combined vote maps

**Figure 3:**
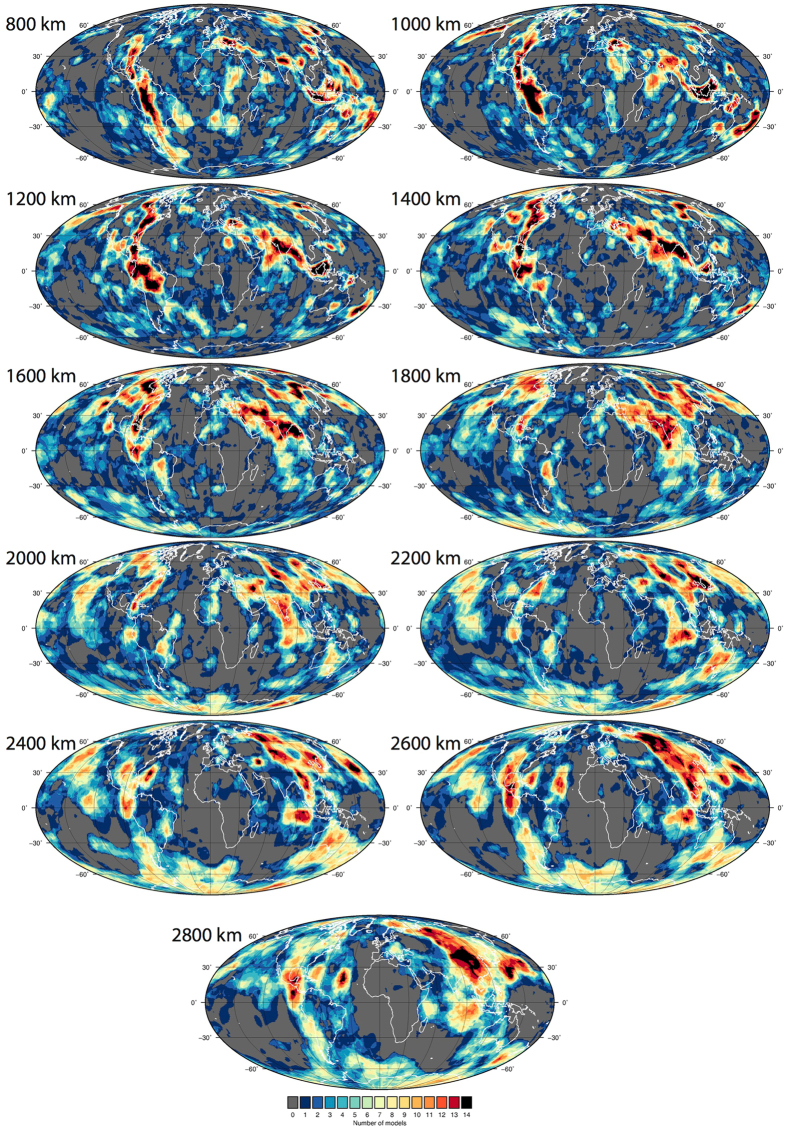
Vote maps for the lower mantle. Vote maps for the reference case (contoured by mean positive value [MPV] and layer depth mean removed [LDM]) from 800–2800 km for the 14 combined models. Figure generated using Generic Mapping Tools (GMT v5.3.1; http://gmt.soest.hawaii.edu/).

**Figure 4:**
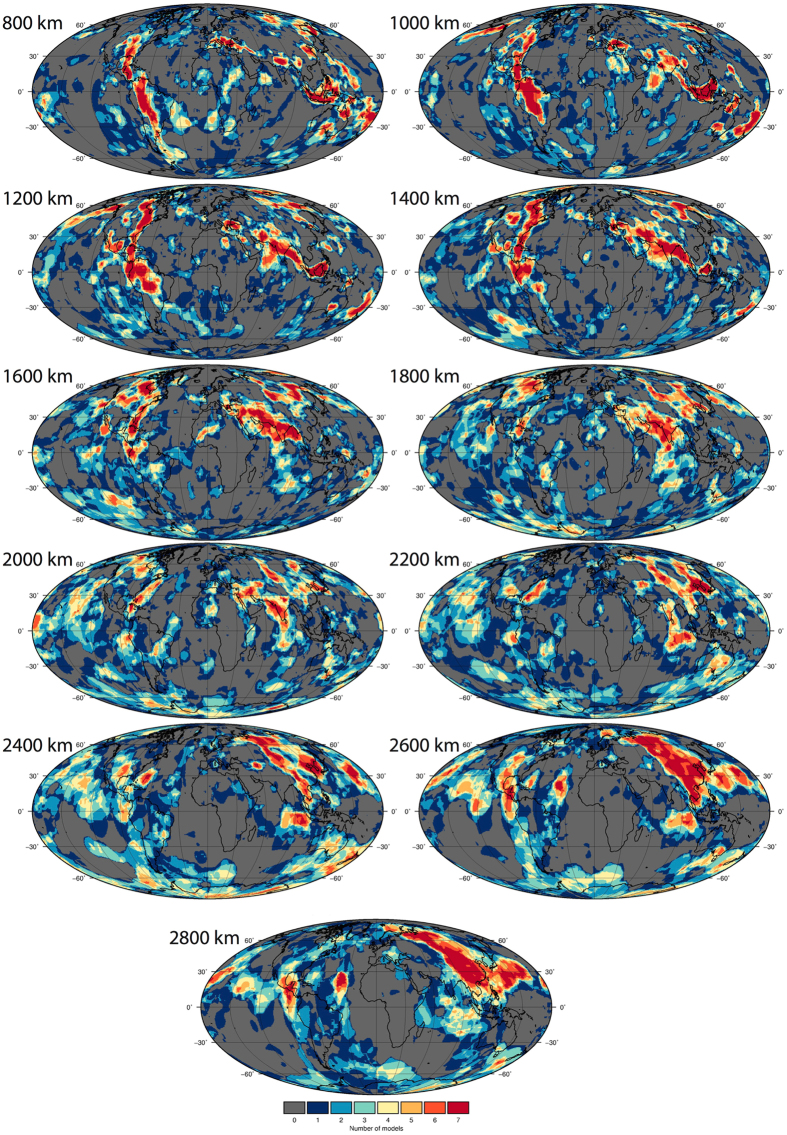
Vote maps for only the combined P-wave models, reference case (as in Fig. 3). Figure generated using Generic Mapping Tools (GMT v5.3.1; http://gmt.soest.hawaii.edu/).

**Figure 5:**
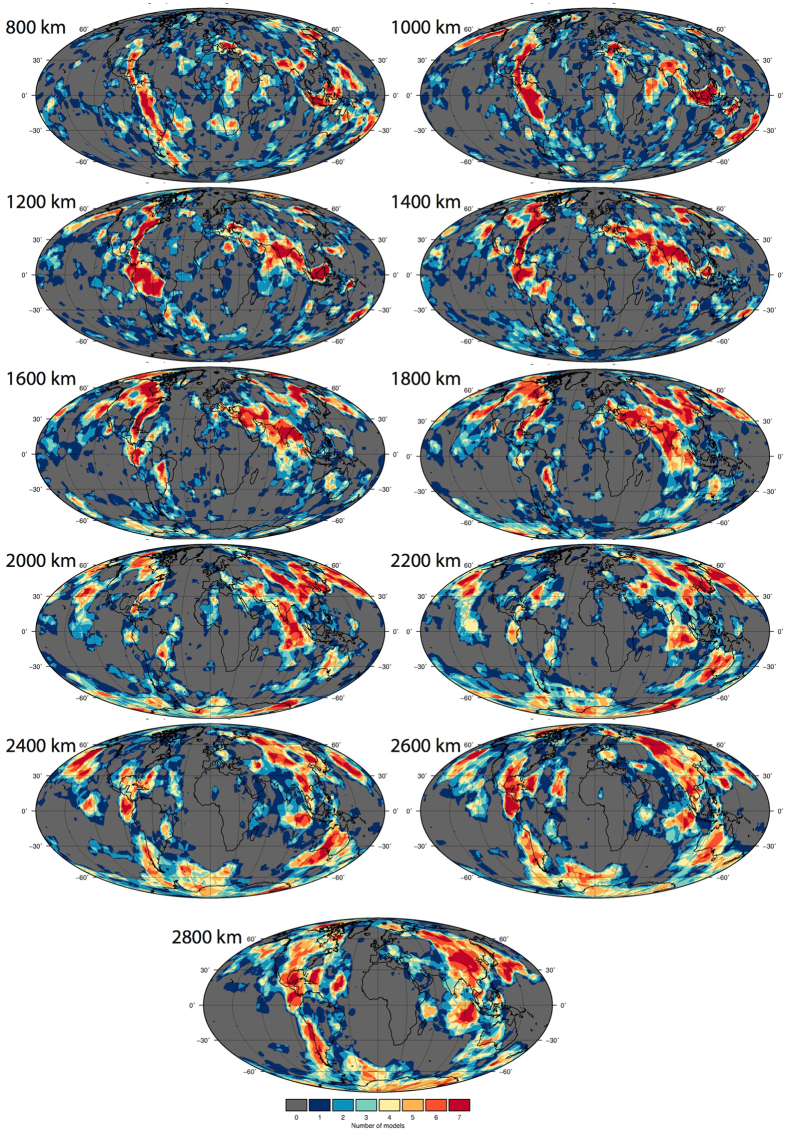
Vote maps for only the combined S-wave models, reference case (as in Fig. 3). Figure generated using Generic Mapping Tools (GMT v5.3.1; http://gmt.soest.hawaii.edu/).

Figure 3 shows the vote maps for the reference case of the 14 combined models, a function of combining the P-wave models (Fig. 4) and S-wave models (Fig. 5) (LDM retained Figure S2; Standard deviation [STD] contour value shown in Figure S5; Root Mean Square [RMS] contour value shown in Figure S6, polar projection Figure S8). The vote maps show that with increasing depth, higher vote regions transition from elongate to progressively longer wavelength aggregate and sub-rounded structures, ultimately portraying the well-known degree-two structure of the lowermost mantle. At 800 km, maximum-vote regions (identified by a 14-vote count only) are imaged under the eastern US, central and northern South America, Mediterranean, India, easternmost Eurasia including near Kamchatka, Southeast Asia and the western Pacific (from east of the Philippines to north of New Zealand). At 2800 km, maximum–vote regions are predominantly restricted to regions under the Americas and eastern Eurasia.

#### Difference between P-wave and S-wave anomalies

**Figure 6:**
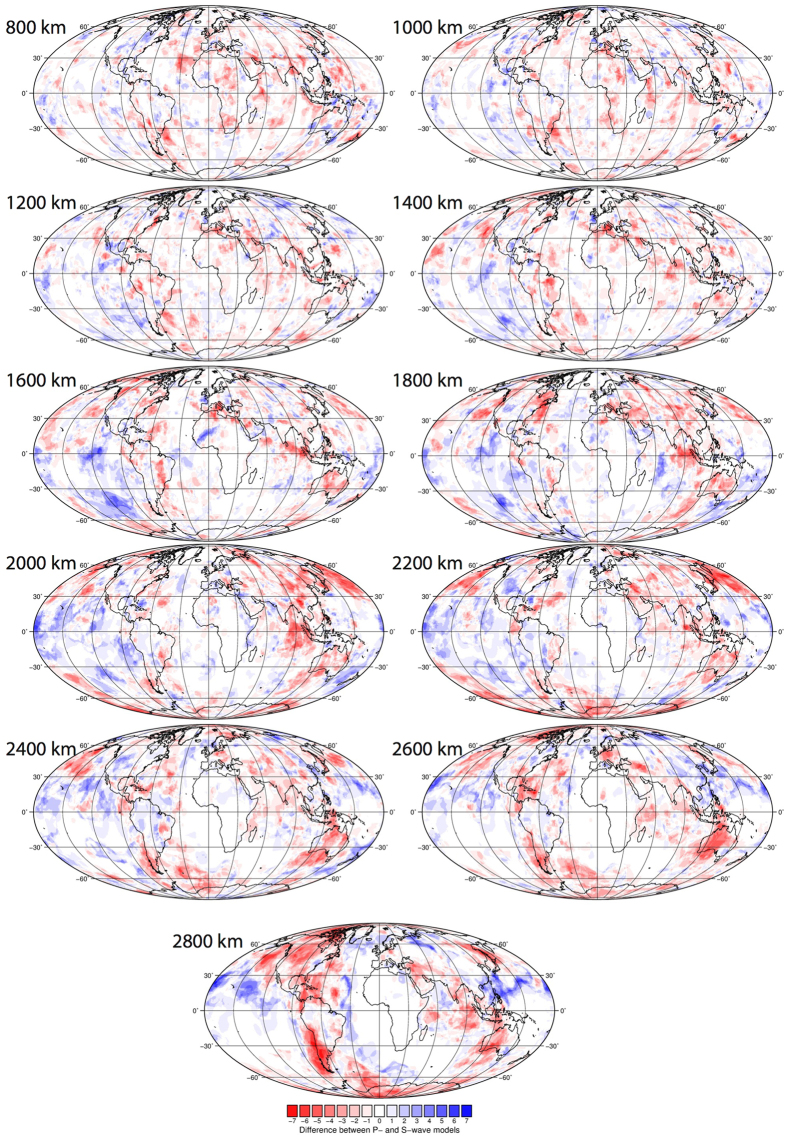
Difference map of the P-wave model vote maps minus the S-wave vote maps, as in Figs 4 and 5. Negative (red) values indicate regions where the S-wave models predict a vote count that is higher (i.e. more agreement between the S-waves) than that predicted in the P-wave models. Vice versa for positive (blue) values. Figure generated using Generic Mapping Tools (GMT v5.3.1; http://gmt.soest.hawaii.edu/).

Figure 6 shows maps generated by subtracting the S-wave votes (Fig. 5) from the P-wave votes (Fig. 4), and spatially illustrates the regions where the two groups differ. The maps generally show that as depth increases, the differences between P- and S-wave votes become more pronounced and the pattern more defined (differences are clustered). The difference maps may highlight the bias that can be made in slab identification by using only one type of dataset, particularly on a regional or depth-restricted scale.

At shallow lower mantle depths, short-wavelength (~100’s km) scattering of difference structures both under the continents and the oceans is observed. By around 1600 km, and below, the pattern becomes more defined. There is significant regional variability throughout the depths, though some are worth noting: S-wave models display high votes under the northern Pacific at 2000–2200 km, Australia from 1600–2800 km, eastern US at 1800 km, central east Atlantic (2000–2800 km; variation also in P-waves), Arctic at 2600 km, and east of Sumatra between 1600–2200 km. Conversely, there is a tendency towards more P-wave votes under the southeast Pacific at 1400–2200 km and northwest Africa at 1600 km. The overall agreement (white/light colours) under central to southern Africa is not unexpected considering the lack of post Paleozoic subduction in the region (plate reference frame of ref. 37). Notably at 2800 km, the S-wave models image a belt of high votes that are not captured in the P-wave models to the same extent, running under the Americas, Antarctica, Australia and southern Eurasia. This pattern matches documented regions of long-lived subduction and may also be related to the LLSVPs (Fig. 3). The P-wave models show a strong positive anomaly band at 30°N running across the Pacific at 2800 km depth that is not as strongly captured by the S-waves.

### Vote maps – maximum agreement and surface area

**Figure 7:**
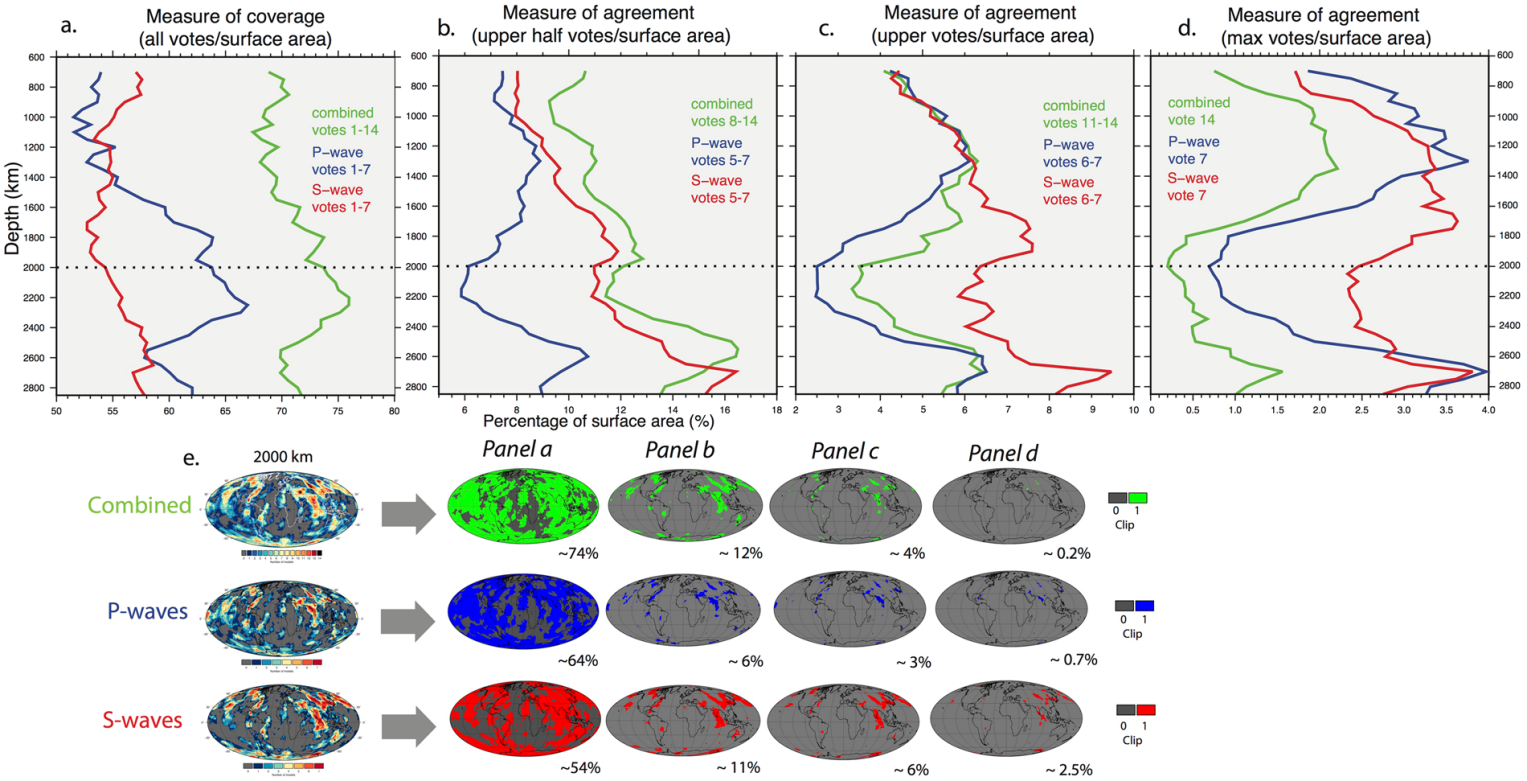
Measure of coverage and agreement of the vote maps as applied to Figs 3–5. (**a**) Area located within the vote maps for all non-zero votes divided by the total area. Combined 14 models in green, blue for P-waves and red for S-waves. (**b**) Area located within the upper half of votes (i.e. 8–14, or 5–7 vote inclusive) divided by the total area (**c**) Area located within the uppermost votes (i.e. 11–14, or 6–7 vote inclusive) divided by the total area (**d**) area of maximum vote count only (i.e. 14/14 for the combined models in green, or 7/7 for the P- and S-waves) divided by the total area (**e**) visual explanation of plots (**a**–**d**) at 2000 km showing original grids (left) and the clipped measure of coverage (right, solid colour). Figure generated using Generic Mapping Tools (GMT v5.3.1; http://gmt.soest.hawaii.edu/).

The vote map characteristics can be quantified based on the calculation of the % surface area of votes as compared to the total surface area (Fig. 7; values listed in Table S3; RMS and STD shown in Figure S7). Figure 7a shows a consideration of all non-zero votes, however, this should be treated with caution because areas of low vote count may actually belong to the “null space”, and can be considered analogous to noise. It follows that the prediction of a large surface area by considering all (non-zero) votes does not necessarily translate to greater agreement when considering higher vote counts, and that the collection of all non-zero votes over predicts the coverage of slabs. The stark differences in profiles from cases with all the non-zero votes (Fig. 7a), the upper half of votes (Fig. 7b), uppermost votes (Fig. 7c), and the maximum agreement case (Fig. 7d) demonstrate the influence of the low vote areas.

When considering only the maximum votes in the combined models (i.e. 14/14 votes, Fig. 7d), surface coverage is greatest at 1350 km (2.2% surface area) and least at 2000 km (0.2%). The P- and S-wave classes follow a similar depth-dependent trend to each other. Notably, for the shallower depths, the maximum agreement (i.e. 7/7 votes) in the P-waves is greatest at 1300 km (3.8%) below which there is a sharp decrease to a minimum agreement at 2000 km (0.7%). For the S-waves, maximum agreement is slightly deeper, at 1700 km (3.6%), and follows a similar, but less pronounced, decrease than the P-waves to a minimum between 2000–2250 km (~2.3%). There is another increase to a secondary peak at 2700 km in both models (~3.8%), followed by a decrease in the lowermost mantle. On average there is slightly less maximum consensus in the P-waves (mean for all depths 2.3%) than the S-wave models (2.8%), with a notable difference between the two models in the mid-lower mantle depths. By extension this analysis suggests that the S-waves image more slabs (in terms of the absolute surface area of the most robust slabs) than the P-waves in the mid lower mantle.

### Vote maps - comparison to subduction flux

**Figure 8:**
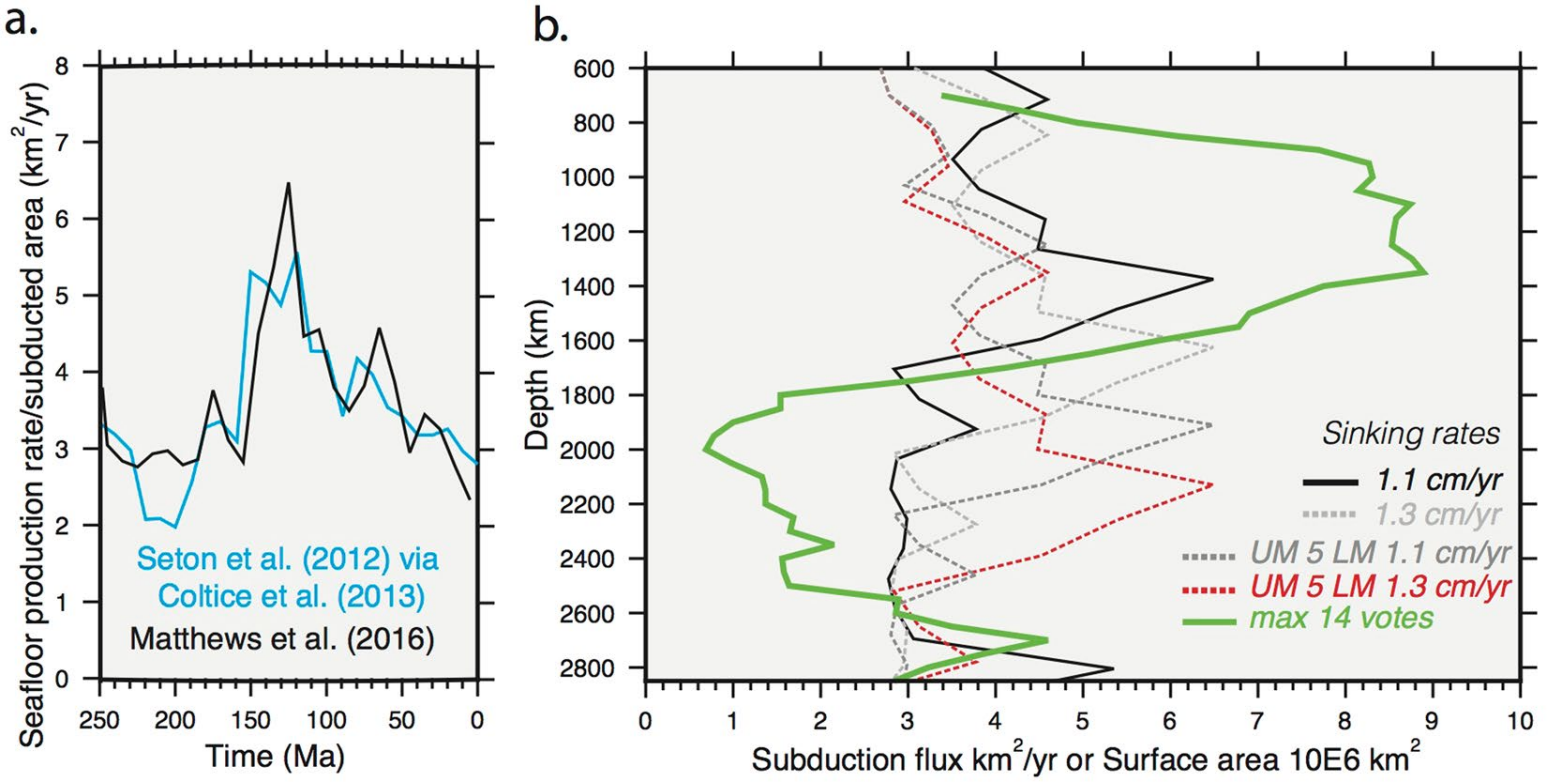
Subduction flux comparison. Panel a. crustal production rates (km^2^/yr) from ref. 39 plate reconstruction in blue, and subducted area (km^2^/yr) from the updated model^37^ in black (**b**). Green line shows depth against the surface area (millions of km^2^) of the maximum votes for the combined models (similar to Fig. 7) and scaled for the radius. Compared against depth-converted subduction flux from panel a as based on alternative sinking rates; black and light grey are globally averaged sinking rates of 1.1 and 1.3 cm/yr respectively, and darker grey and red are faster alternatives which assume 5 cm/yr in the upper mantle and 1.1 and 1.3 cm/yr in the lower mantle, respectively.

The surface area results for maximum votes (here in millions of km^2^ instead of % surface area) were scaled by the equivalent radius of the depth slice (Fig. 8b, green line). The trends of the individual wave groupings are comparable to those for Fig. 7d and only the combined models are shown for simplicity. An increase in the amount of surface area contoured is observed between 700–1400 km depth (~3 to 8 million km^2^), a decrease from 1400–2000 km (~1 million km^2^), followed by an increase to a second maximum at 2700 km (~5 million km^2^) and a decrease towards the core-mantle boundary (~3 million km^2^).

Figure 8a shows a comparison of the subduction flux as determined from the seafloor production proxy (refs 38, 39) and as calculated directly from the plate reconstruction model of ref. 37. To the first order, the two curves are similar and thus we focus on the recent plate reconstruction model (Fig. 8b). We are limited to discussing surface area rather than volume, however, it is worth noting that the surface area of the 14 votes (green line) and subduction flux curves (black, grey and red lines) are of the same magnitude; between 1000–1400 km the maximum vote surface area is larger by around 3–4 million km^2^ (the equivalent size of India). Conversely, between 1800–2600 km the vote values are around 2 million km^2^ less than modeled by the subduction flux. In terms of the trends, using the whole mantle average sinking rates (1.1 and 1.3 cm/yr), Panel 8c shows a reasonable first order match between the maximum vote curve and the subduction flux curve. A sinking rate of 1.1 cm/yr (black line) matches the best, including the peak in area/rates around 1400 km, the pronounced decline around 1600–1800 km and the increase below 2400 km. However, when using the age-depth conversion with a faster average sinking rate in the upper mantle (5 cm/yr; dark grey and red line) than for the lower mantle (1.1 and 1.3 cm/yr), the match between the depth/age changes in the maximum vote curve and the subduction flux are almost opposite, notably so between 1800–2600 km depth.

## Discussion

The vote maps presented here are only as robust as the individual tomography models which they are comprised of, and thus the varying degrees of overlap of data input and parameterization renders the votes somewhat biased as they are not truly independent. Bias could also be manifest in a regional/depth sense due to resolution and model regularization^21^, which can be different between tomography models. Furthermore, the absence or presence of a high or low vote count, or “agreement”, does not necessarily mean that one suite of models are better or more robust than the other; a high vote could reflect bias in the same data input. Nonetheless, a maximum vote count of 14 out of 14 for widely used, global tomography models, including both P- and S-waves, provides some of the strongest evidence for subducted material. We are confident that the maximum vote class, i.e. 14/14, or 7/7 models to a lesser extent, represents the most robust slabs and illustrates key depth-dependent trends—we herein refer to these highest MPV contour votes as “slabs”.

Due to our removal of the LDM, the location of the MPV for any given model and depth is a function of the full distribution of wavespeeds at that depth, including negative wavespeeds. For example, for a negatively-skewed distribution (such as the lowermost mantle), the LDM will be less positive than the median wavespeed value, such that removal of the LDM will result in a larger number of wavespeed values being classified as “positive”, with respect to a normal distribution of like-variance. However, this would also result in a relative increase in the calculated MPV, partly mitigating the inclusion of possibly “null-space” wavespeed values. The variance of the full wavespeed distribution also plays an important role, as the calculated MPV will become more positive with increasing variance. The common reduction in the variance of the wavespeed distribution of most models in the mid-lower mantle thus partly explains the observed decrease in the MPV at these depths, whereas the increased variance and negative skewness of the distributions of many of the models in the lowermost mantle explains the pronounced increase in their MPVs at those depths. Because of these competing effects, it is not straightforward to interpret a changing MPV as a direct measure of the changing volume of slab material in the mantle – in fact their trends are largely decoupled – and it is for this reason that we use a voting map to infer true slab volume changes. Nevertheless, the depth-dependence of the MPV presents a simple and useful measure of the changing wavespeed distributions, for example clearly expressing a strong effect of the LLSVPs on the S-wave models in the lowermost mantle.

On average, while the MPV calculated from the P-wave models is generally lower than that of the S-wave models (Fig. 2a), this does not translate to a significant difference in area contoured by the MPV (% or absolute surface area Fig. 2b,c) due to the P-wave amplitudes being lower (more restricted). Most of the variability between individual tomography models illustrates a function of data input and parameterization methods. While a larger area of non-zero votes is observed in the P-wave models (relative to the S-waves; Fig. 7a) in the mid-lower mantle, this trend does not persist in higher vote count considerations (see below), and we consider it insignificant. However, this highlights an important point, namely that the specific MPV threshold is arbitrary and only used as a filter with which to concentrate potentially meaningful votes. This means that some wavespeed values that belong to the “null space” will pass through the MPV filter, contributing noise to the analysis. The voting process should highlight these features, which, if not associated with a significant positive wavespeed value, should not consistently appear across the bulk of model votes, and will therefore present as low vote regions. We can therefore consider low vote regions as noise. It follows that a depth with a large non-zero vote area (i.e. 1–14 votes) does not necessarily translate to a depth with great agreement; rather the highest vote counts should be considered (i.e. 14 votes), which is a more appropriate measure of the presence of robust slabs.

This effect is further demonstrated in the disparity between the depth profiles of the P- and S-waves when considering all non-zero votes, the upper half of votes, uppermost votes, and only the maximum votes (Fig. 7). To this end, our combination of the MPV contour value and the use of 7 or 14 models is a controlling factor on the total surface area measured for the votes. It suggests that considering all non-zero votes rather than just the maximum votes in isolation is misleading, and that the analysis should either be further restricted to at least (the upper) half of the number of models, or ideally only the maximum votes when looking at depth-dependent trends. This indicates that, generally, the more individual tomography models used in the study, the more robust is the analysis based on the MPV contour. In other words, if using fewer models, the match between them could be based on a more restricted/higher contour threshold e.g. RMS or upper third quartile. A comparison with the results from using the less positive STD metric or more positive RMS metric hold similar results to those described, albeit of a magnitude shift as expected from the threshold value.

To the first order, the P-wave and S-wave vote maps image a similar distribution and amount (surface area, within 3%) of positive wavespeed anomalies (Figs 4 and 5), and exhibit comparable depth-dependent trends. The main difference between the classes for the maximum vote case is between 1400–2300 km depth (Fig. 7), where the S-waves predict a larger coverage. A minimum in correlation (in terms of coverage) between P- and S-wave at 2000 km has also been noted in other studies^23^. This difference is possibly due to ray coverage or compositional changes (e.g. ref. 40). It could also be related to S-wave resolution, leading to an apparently larger slab expression, though we note that such an effect might be expected to be seen across all depths and not just the mid-lower mantle. Nonetheless, mid-mantle depths generally exhibit the lowest P- and S-wave amplitudes (Fig. 1). Notably, this highlights the potential under/over prediction of slabs when only using the P/S-wave cases, respectively.

Our analysis of the surface area of the maximum vote counts for the combined case (Fig. 7d) reveals that the upper part of the lower mantle between ~1000–1400 km shows the greatest coverage of maximum agreement, or potential slabs. This effect is also seen in the uppermost votes (Fig. 7c), which might be more appropriate for considering slab geometries (as the maximum votes are very restricted in overall coverage, see panel e). Slab thickening by a factor of 2–3 upon entering the viscous lower mantle is generally expected^41^. A change in spectral character of seismic wavespeeds from the upper to lower depths of the lower mantle has also been noted, whereby fast anomalies dominate above about ~1500 km and slow anomalies dominate below^42^. This lead ref. 43 to identify a “mid lower mantle transition zone” from around 1200–1600 km.

The increase in maximum slab agreement/coverage is followed by a decrease in the lower half of the lower mantle towards 2000 km. This effect is also seen in the half and uppermost vote panels (Fig. 7b,c). A depth with a smaller area of maximum agreement, such as between 1800–2600 km depth could indicate a range in which there truly are fewer slabs but could alternatively reflect a decreased ability of tomography to accurately resolve true slab features. Thermal diffusion and dissipation mean that slabs in the lower mantle appear more smeared than those in the upper mantle. This is also compounded by limited data coverage, leading to relatively coarser image resolution and discrepancies across tomographic models in the lower mantle, even for large-scale features^24^. Unfortunately, with the slab vote map methodology it is not possible to rigorously distinguish between these scenarios (i.e. true slab volume or seismic resolution), but by considering changes in the apparent area of slabs with the use of less stringent vote criteria it is possible to infer what may be real depth-dependent slab volume changes. Accordingly, the similar depth-dependent trends observed between the upper votes and maximum votes suggest that there may be a true reduction in slab volumes in the mid-lower mantle relative to the upper-lower mantle above.

In the case of the lowermost mantle, the wavespeed distributions are generally negatively skewed, due to the strongly negative seismic wavespeeds associated with the LLSVPs, and the variance is greater, together leading to a general increase in the calculated MPV. In this respect, the dominance of the LLSVPs appears to promote the strong imaging of “slabs”, as otherwise ambient mantle, now spatially confined to the area outside the LLSVPs, is more likely to be classified as “positive”. This is reflected in the increased agreement at around 2600 km in both classes of models (Fig. 7). Furthermore, when the maximum agreement is changed to the upper half of models (i.e. 8–14 or 5–7 inclusive count) the S-waves show a particularly pronounced dominance over the P-waves in the lower half of the mantle because they are strongly affected by the presence of the LLSVPs. Due to the restriction of ambient mantle to the same common areas outside the LLSVPs, votes coming from the “null space” may still reach a high vote count, and could be difficult to distinguish from the “true” slab signal. Thus, increased caution is warranted for any attempt to image and interpret slabs below 2600 km based on the vote maps.

While the accuracy of the tomographic imaging (spatial resolution and model amplitude errors) is inherently critical in this analysis, a possibility is that these depth-dependent slab volume changes are a reflection of true changes in the time-dependent mass flux to the mantle, especially for depths shallower than those occupied by the LLSVPs (i.e. shallower than the few hundred kms above the core-mantle boundary). The first order match in magnitude of the surface area of the maximum 14-vote slabs and subduction flux estimates suggests that the use of the maximum vote criterion may allow an appropriate measure of depth-dependent trends in subducted slab material. We note that both estimates, however, lack the third dimension of depth (to provide volume). Considering a more generous threshold e.g. 11/14, which also follows a similar depth-dependent trend, would be more appropriate in considering actual slab contours. The magnitudes of the depth-dependent changes in surface area are higher for the individual P- or S-wave cases (not shown on the scale of Fig. 8b), illustrating the effect of using 7 over 14 models.

With the application of a globally averaged mantle sinking rate to the independent subduction flux curve, a direct comparison with the depth-dependent vote maps results can be made. Use of a sinking rate of ~1.1 cm/yr shows a good first-order match between these trends. If a faster upper mantle sinking velocity of 5 cm/yr is considered, and with no slab stagnation in the transition zone, a near anti-correlation is observed, requiring an alternative explanation for the slab area depth-dependence. The independent observation of slab stagnation or thickening at ~1000–1500 km has been attributed to a smooth density and/or viscosity change (e.g. refs 43 and 44). This effect may be partly driven by Fe++ spin transition^45, 46^, an intrinsically dense lower mantle component (subducted MORB)^47^, or some (other) thermo-chemical transition^48^. Such a transition may cause flattening, fragmentation and thermal dissipation of the slabs at these depths, therefore leading to an apparent increase in slab area (volume) (e.g. refs 42, 43, 49 and 50). Observed mid-mantle seismic reflections^51, 52^ may also be related to subduction related features but require further analysis.

**Figure 9:**
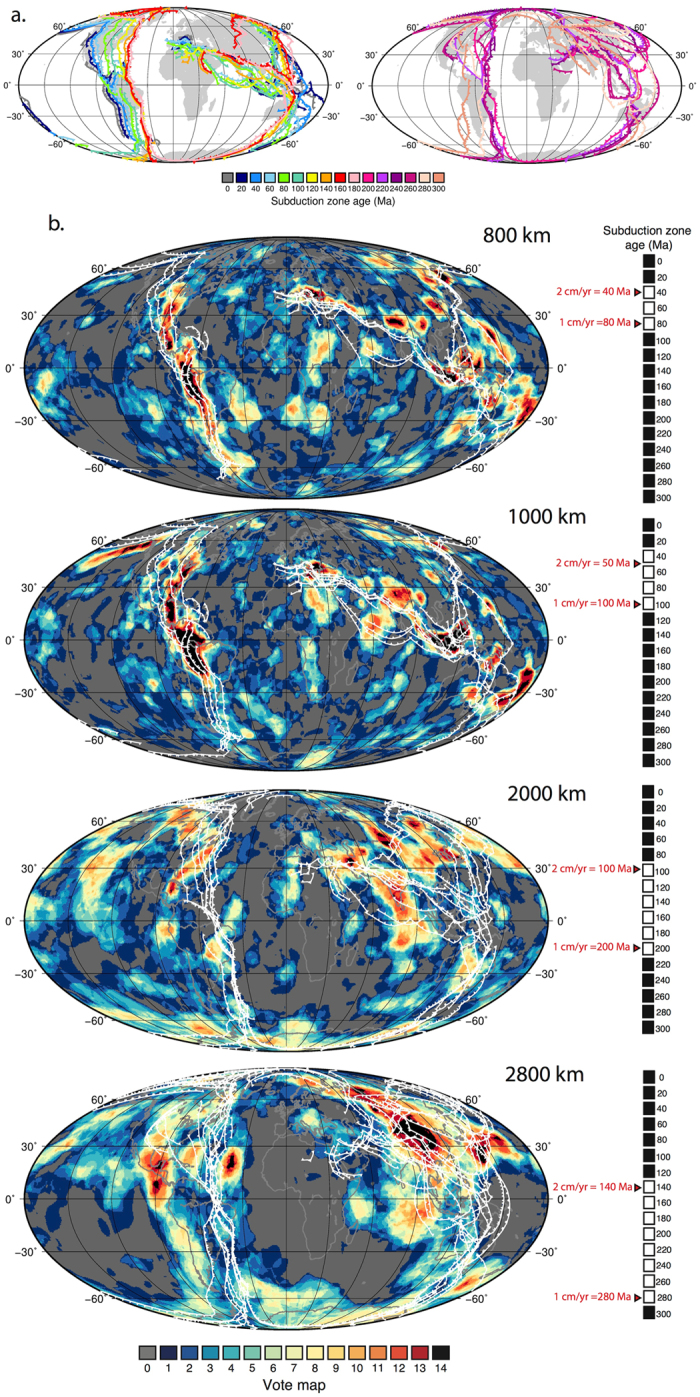
Location of subduction zones at the surface as compared to the combined vote maps for selected depths. Panel a) subduction zone locations based on ref. 37 coloured in 20 Myr increments. Panel b) shows vote maps at 800, 1000, 2000, 2800 km depth superimposed with a selection of subduction zones (white) as based on two end member average sinking rates of 1 and 2 cm/yr (black and white scale to the right hand side). Figure generated using Generic Mapping Tools (GMT v5.3.1; http://gmt.soest.hawaii.edu/).

Present-day subduction zones exhibit a dichotomy between long linear subduction zones, such as those around the circum Pacific (Fig. 9a), and smaller more isolated subduction zones, such as those in Southeast Asia and the Mediterranean. This reflects an interplay between plate velocities, oceanic basin size, lithospheric structures and age, trench motion and curvature, and intra-oceanic versus continent-proximal settings, among other factors (e.g. ref. 53). The combination of both broad and short subduction zones is also presented back in time (Fig. 9a,b), however, we note that decreasing constraints will simplify reconstructed subduction zone lengths and geometries. The sizes and shapes of the maximum vote slabs are highly variable (Fig. 3); some of the slabs show a lateral linearity, for example under North America, which suggests long-lived and contiguous subduction zones. Others are smaller, sub-rounded or patchy which might be attributed to shorter lived, isolated, or strongly migrating subduction zones, including in intra-oceanic or back-arc settings. While key Cretaceous-Jurassic plate tectonic events such as fluctuating ridge activity in Panthalassa and the breakup of Pangea^37^ may explain the slab distribution shown here, we do not speculate further (Fig. 9).

## Conclusions

Our vote map technique is neither a measure of the existence of an actual slab nor a critique of individual tomography models. The quality of vote maps is highly dependent on the independence of the tomographic models as implicitly defined for each model by the data inputs, the model parameterization used to construct the tomography model, the attained resolution and model amplitude error, and assumptions regarding data errors and model regularization. Nonetheless, the vote maps provide a useful indication as to the distribution of the most robust slabs that are imaged across a selection of 14 tomography models. We show that these remnants of subduction can be identified through the use of a model- and depth-dependent threshold metric, together with a voting approach.

The use of alternative threshold metrics produce similar depth-dependent results. On average 20% of a depth’s surface area is contoured by the MPV but only 1% is in maximum agreement when considering 14 models. A peak in the amount (coverage and agreement) of the most robust slabs is identified between 1000–1400 km, and a minimum between 1400–2500 km depths. These trends may match an independent measure of subduction flux using an average mantle sinking rate of 1.1 cm/yr. The use of a faster rate yields an anti-correlation between the subduction flux and our results, and may indicate that the observed depth-dependent slab volume changes are due to slab stagnation, either in the upper mantle transition zone, or in the mid-lower mantle where there may be a viscosity increase. On average, the S-wave models generally agree more than the P-wave models, whilst noting that the P- and S-wave models are parameterized differently, and that S-waves have a lower sensitivity to the detail of mantle structure. The identification of slabs in the lowermost mantle, below 2500 km, is likely to be greatly complicated by the presence of the large, antipodal LLSVPs and thus interpretations derived from the lowermost mantle should be treated with caution. Our vote maps constitute an open-source workflow and can be added to and refined with advances in seismology and geodynamics.

## Methodology

For this study we analysed a total of 14 global seismic tomography models, split between 7 P-wave and S-wave models each (Table 1). We do not filter the models to exclude any spherical harmonic degrees. Because we are interested in the relative, depth-dependent velocity structure of the models which were constructed against different 1-D reference models (Table 1), we remove the layer-dependent mean (LDM) from each model. In other words, removing the mean permits a discussion that is not dependent on the reference model used to construct the models (Figures S1–S3). We also include a comparison in which all tomography models have been recalculated with the same background model, noting that regularization, ray path geometry and event positions depend on the reference model initially chosen. Nonetheless, for our purposes we find that the use of a background model has a negligible effect (Figure S4).

**Figure 10:**
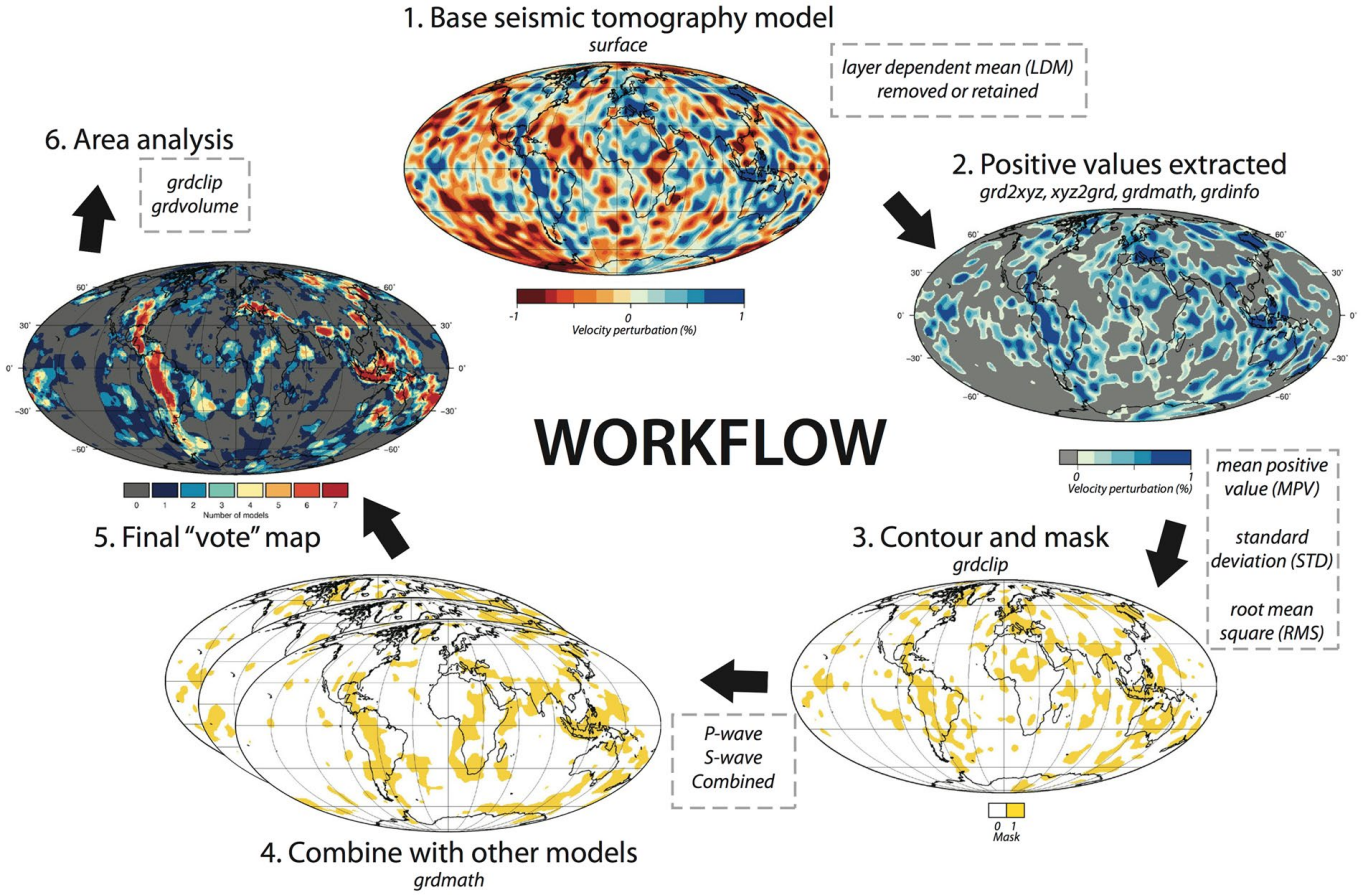
(Methods section). Summary of vote map workflow. Parameter choices are shown in dashed boxes at relevant steps, and Generic Mapping Tools (GMT v5.3.1) commands used are italicized. Panels 1–3 based on S40RTS at 800 km. Figure generated using Generic Mapping Tools (GMT v5.3.1; http://gmt.soest.hawaii.edu/).

Here we focus on the lower mantle and so only consider depths greater than 700 km. Regular grids (0.5°cells) in depth increments of 50 km are derived from the raw tomography models by linear interpolation from the original grids (Table S1). We used Generic Mapping Tools (GMT, version 5.3.1)^54^, and Fig. 10 shows a summary of the methodology and the GMT commands utilized.

The choice of a contour value (% δlnVs, % δlnVp; seismic velocity perturbation) to represent a subducted slab can be derived from a fixed value (e.g. +0.3%), or one that depends on depth- and model-dependent characteristics, which we consider here. For each model (described above; LDM removed and depth interpolated), we extract the positive values at each depth and calculate the mean positive value (MPV to distinguish from the LDM). Areas with wavespeeds in excess of the MPV are then classified as a slab “vote.” Two alternative threshold statistics, the standard deviation (STD) and root mean square (RMS), are shown in the supplementary (Figures S5–S7) and do not significantly change the overall results. To summarize, our reference cases shown herein have the LDM removed, are contoured based on the MPV, and are based on depth-interpolated grids.

The resulting grids can then be added across the different models to generate a vote count, with higher counts signifying greater agreement on any given positive wavespeed feature. Here we present vote maps for the 7 P-wave models and 7 S-wave models individually, and the 14 models combined. To quantify differences in the vote counts between depths and the two classes of tomography models, the percentage of surface area of the respective votes can be calculated. There are several ways of quantifying both the coverage and the agreement between the tomography model groupings, depending on the vote that is considered (e.g. all non-zero votes, or the maximum vote, or a selection thereof), and the reference area (e.g. total depth’s surface area, or all non-zero votes etc). For simplicity, the results are presented as % surface area with respect to the given depth horizon and thus are scaled to absolute area (depth conversion is shown in Section 2.4). We illustrate the effect of choosing only the maximum votes (i.e. 14/14 or 7/7 votes only), or a relaxed selection with lower vote counts included.

A comparison between the scaled surface area of the maximum vote counts and two independent measures of subduction flux derived from two plate reconstructions is also undertaken. The first is a subduction flux “proxy”^69^, whereby seafloor production rates^38^ derived from the plate reconstruction in ref. 39, measure the amount of crustal accretion at mid-ocean ridges from 200–0 Ma. The second directly measures subducted seafloor area based on the recently updated global plate reconstruction of ref. 37. Because the rates from the two subduction flux curves are presented in age (Ma) versus rate (km^2^/yr), they can be converted to depth based on a mantle sinking rate. To satisfy a range of proposed slab sinking speeds we present a first set based on an average whole mantle sinking rate of 1.1 and 1.3 cm/yr in line with global studies (e.g. refs 5, 7 and 8). However, plate convergence rates, typically higher than 1–2 cm/yr have also been used to approximate upper mantle sinking rates^70, 71^ so to satisfy an upper range of speeds we also apply the sinking rates assuming that a slab has already reached 700 km just 14 Myrs after subduction (5 cm/yr).

### Data availability

Datasets for the vote maps, or any additional information can be requested by emailing the corresponding author Grace Shephard at g.e.shephard@geo.uio.no. Data also provided at http://folk.uio.no/gracees/Shephard_SlabVoteMaps/ Additional vote map figures can be generated at. http://submachine.earth.ox.ac.uk. All figures in manuscript generated using Generic Mapping Tools (GMT v5.3.1; http://gmt.soest.hawaii.edu/).

## Electronic supplementary material

Supplementary Material
