# Supplementary material for: On the consistency of seismically imaged lower mantle slabs
Source: Sci Rep. 2017 Sep 8;7:10976. doi: 10.1038/s41598-017-11039-w (PMC5591187; doi:10.1038/s41598-017-11039-w)
Supplement: Supplementary file 1 — Supplementary Material [file 41598_2017_11039_MOESM1_ESM.pdf]

Supplementary material for

"On the consistency of seismically imaged lower mantle slabs"

G.E. Shephard, K.J. Matthews, K. Hosseini, M. Domeier

Nature: Scientific Reports

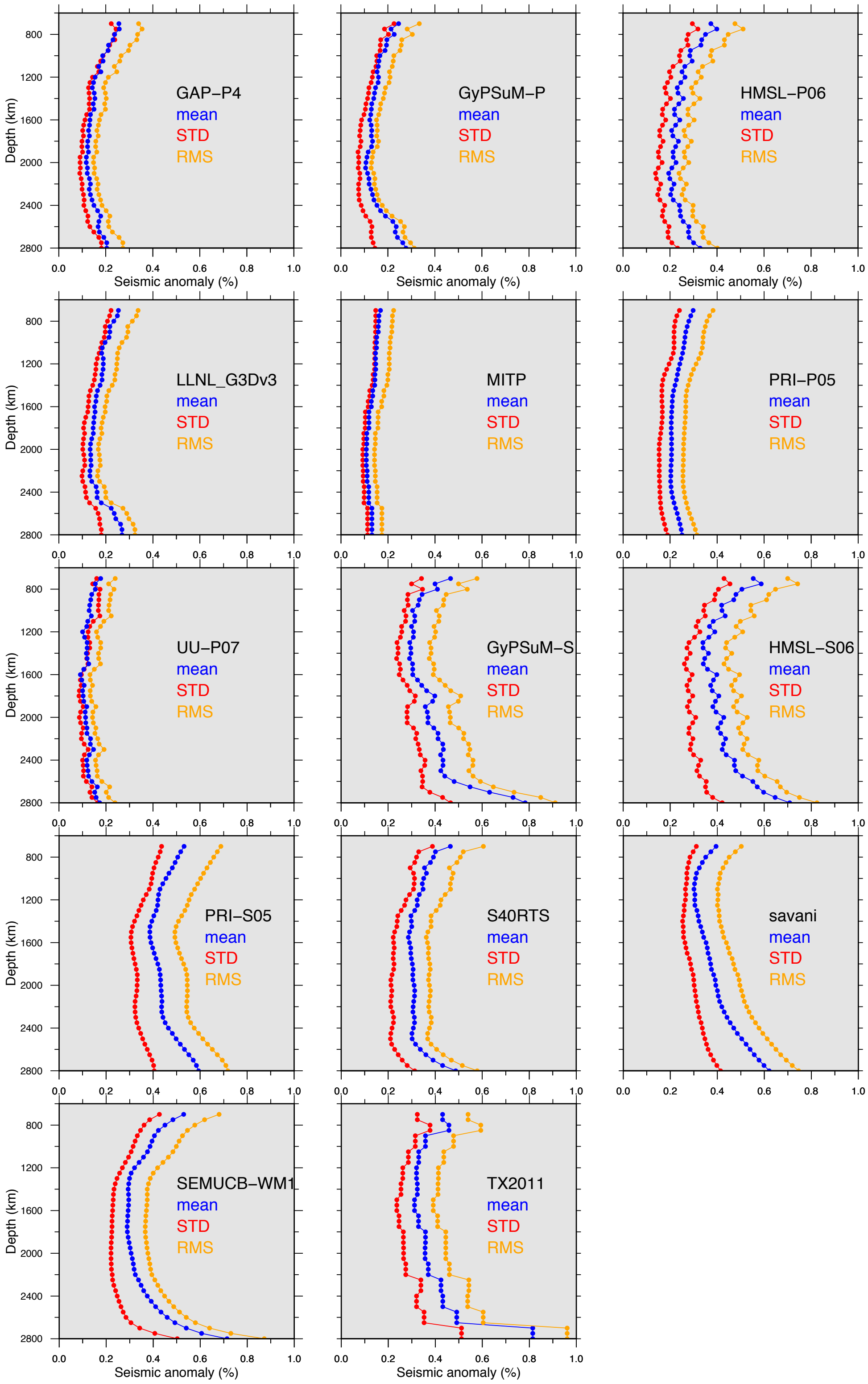

Figure S1. Values of statistical mean positive value (MPV), standard deviation (STD) and root mean square (RMS) calculated for each tomography model at each interpolated depth increment for the positive only values. Generally the

STD values are lower than for the MPV, though exceptions occur especially at shallower depths. The RMS values are consistently higher than for the MPV.

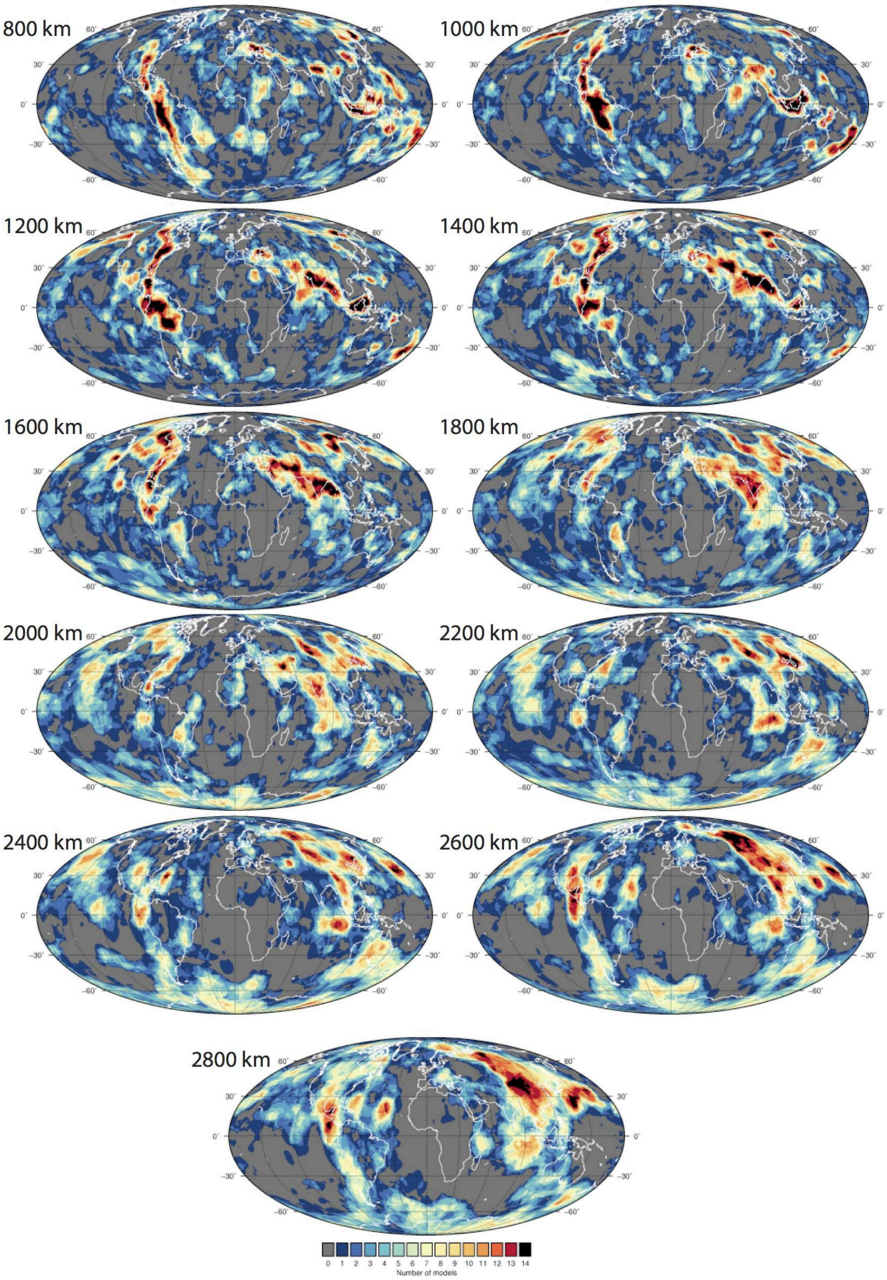

Figure S2. Vote maps from 800-2800 km for the 14 combined models. Grids do not have the mean removed (LDM retained). Note that several models (GAP-P4, SEMUCB-WM1, LLNL_G3Dv3) were not supplied with the means retained, thus this analysis is not completely independent of the reference case results (LDM removed). Figures generated using Generic Mapping Tools as in Figure 10 (GMT v5.3.1; http://gmt.soest.hawaii.edu/).

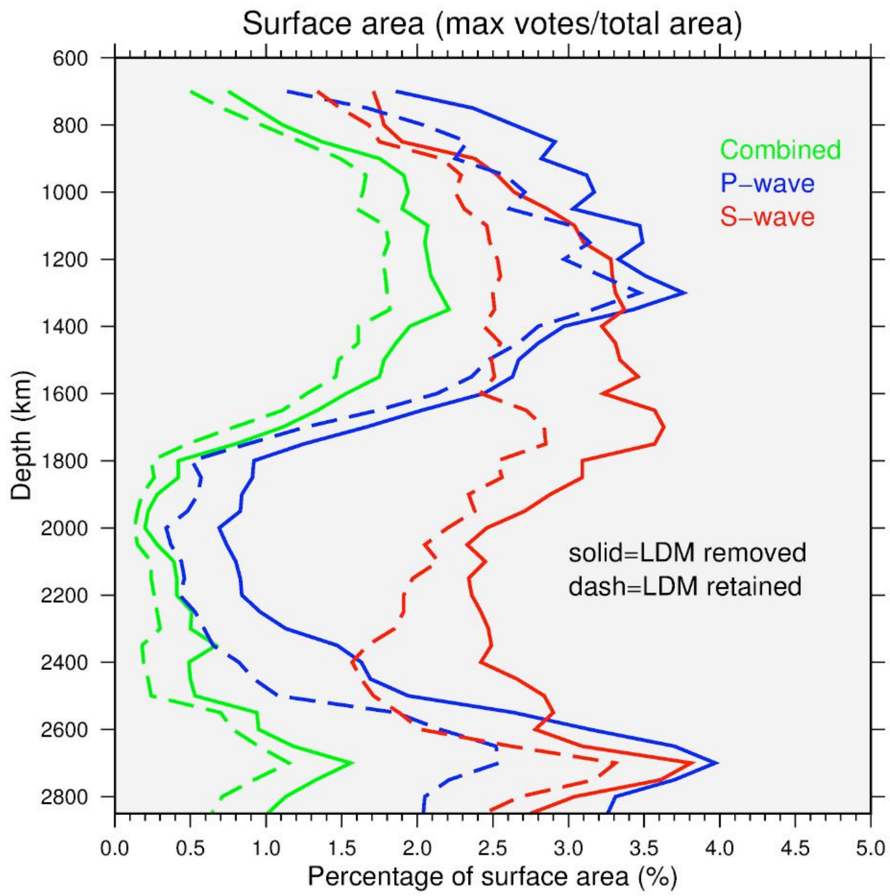

Figure S3. Comparison of LDM retained or removed; Measure of coverage and agreement of the MPV (LDM removed) compared to the MPV derived from the LDM retained grids, dashed lines. Results for the LDM-retained case follow similar depth-dependent trends to the reference case with the LDM-removed, and are of a smaller magnitude.

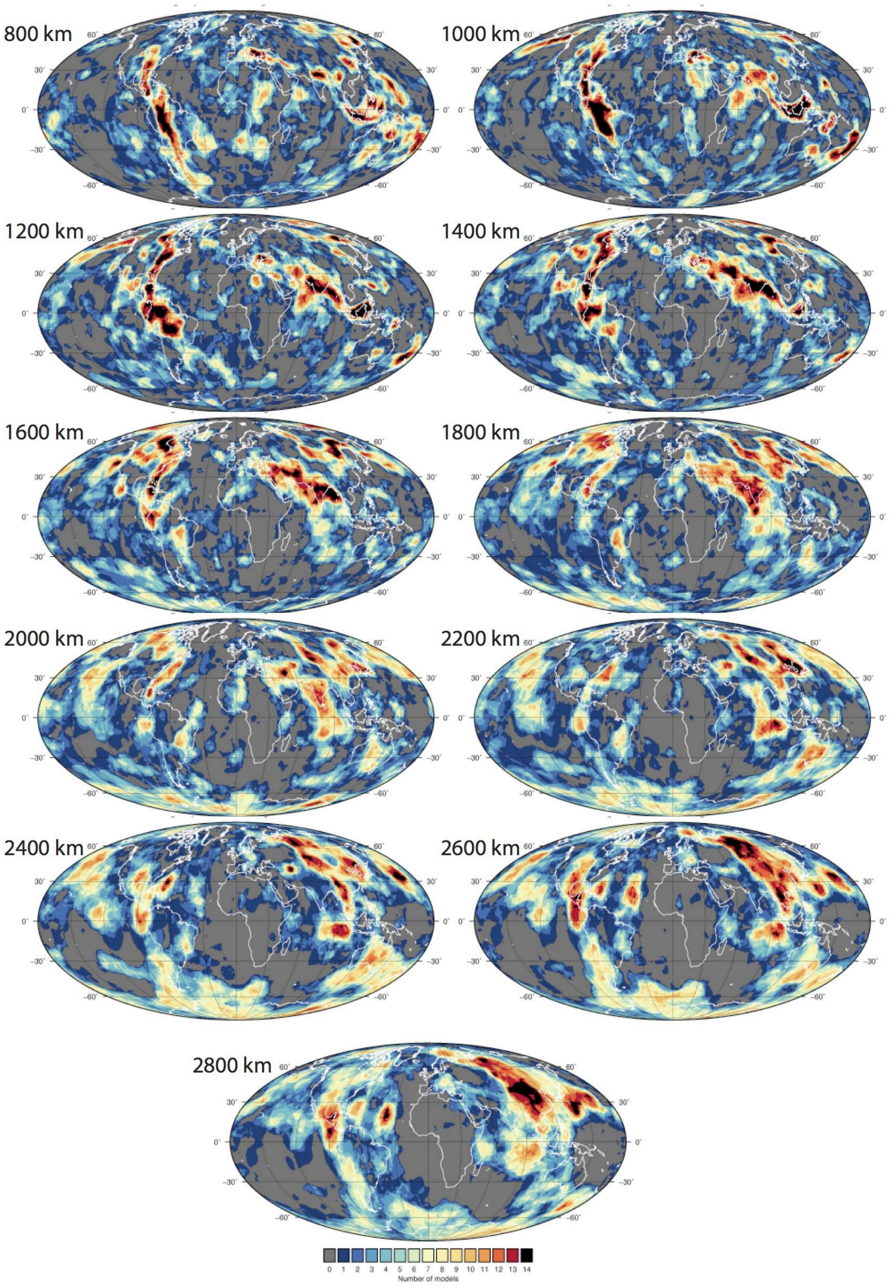

Figure S4. Vote maps from 800-2800 km for the 14 combined models which have been generated with respect to reference model of PREM (Dziewonski and Anderson, 1981). Figures generated using Generic Mapping Tools as in Figure 10 (GMT v5.3.1; http://gmt.soest.hawaii.edu/).

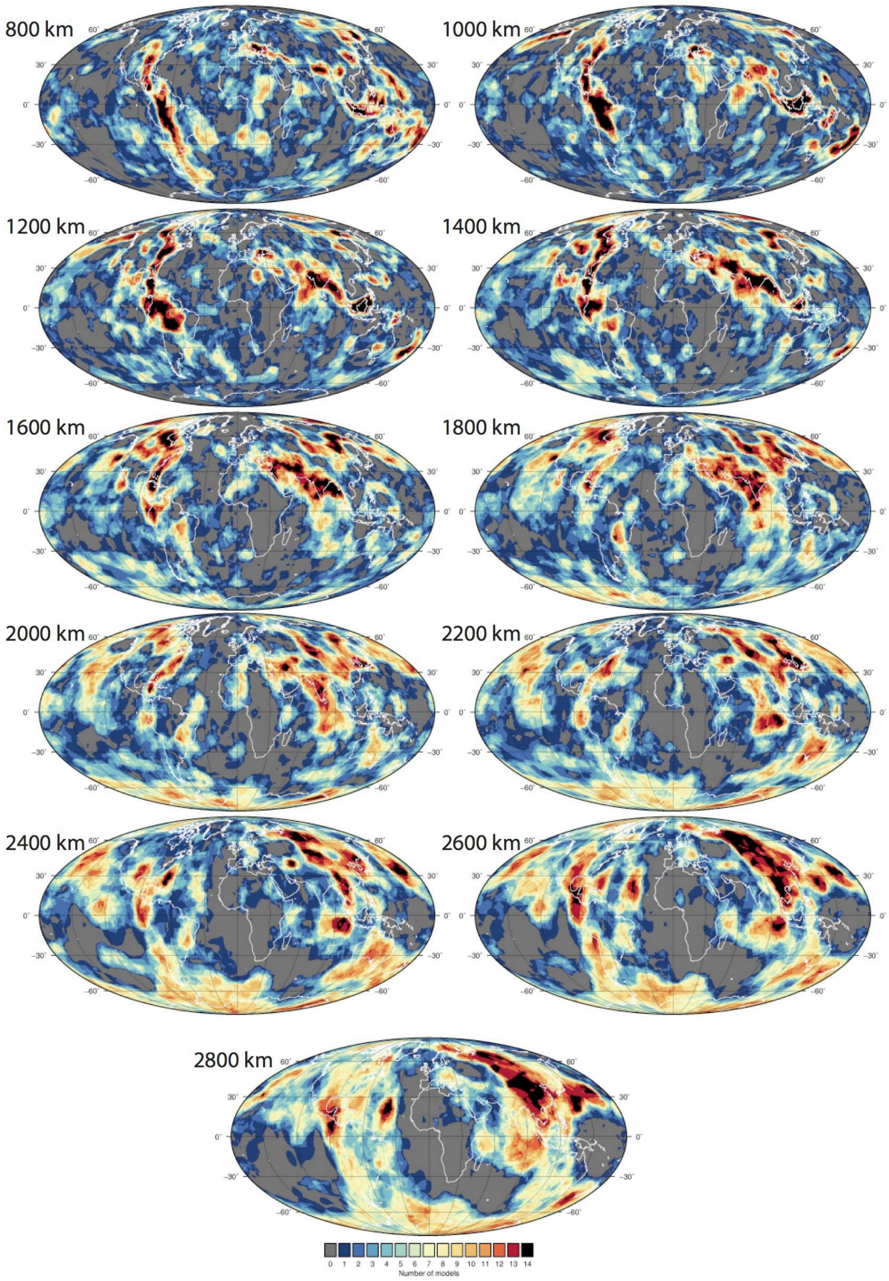

Figure S5. Vote maps from 800-2800 km for the 14 combined models. Grids have the LDM removed and the slab contours are based on the statistical standard deviation (STD) instead of the MPV. Figures generated using Generic Mapping Tools as in Figure 10 (GMT v5.3.1; http://gmt.soest.hawaii.edu/).

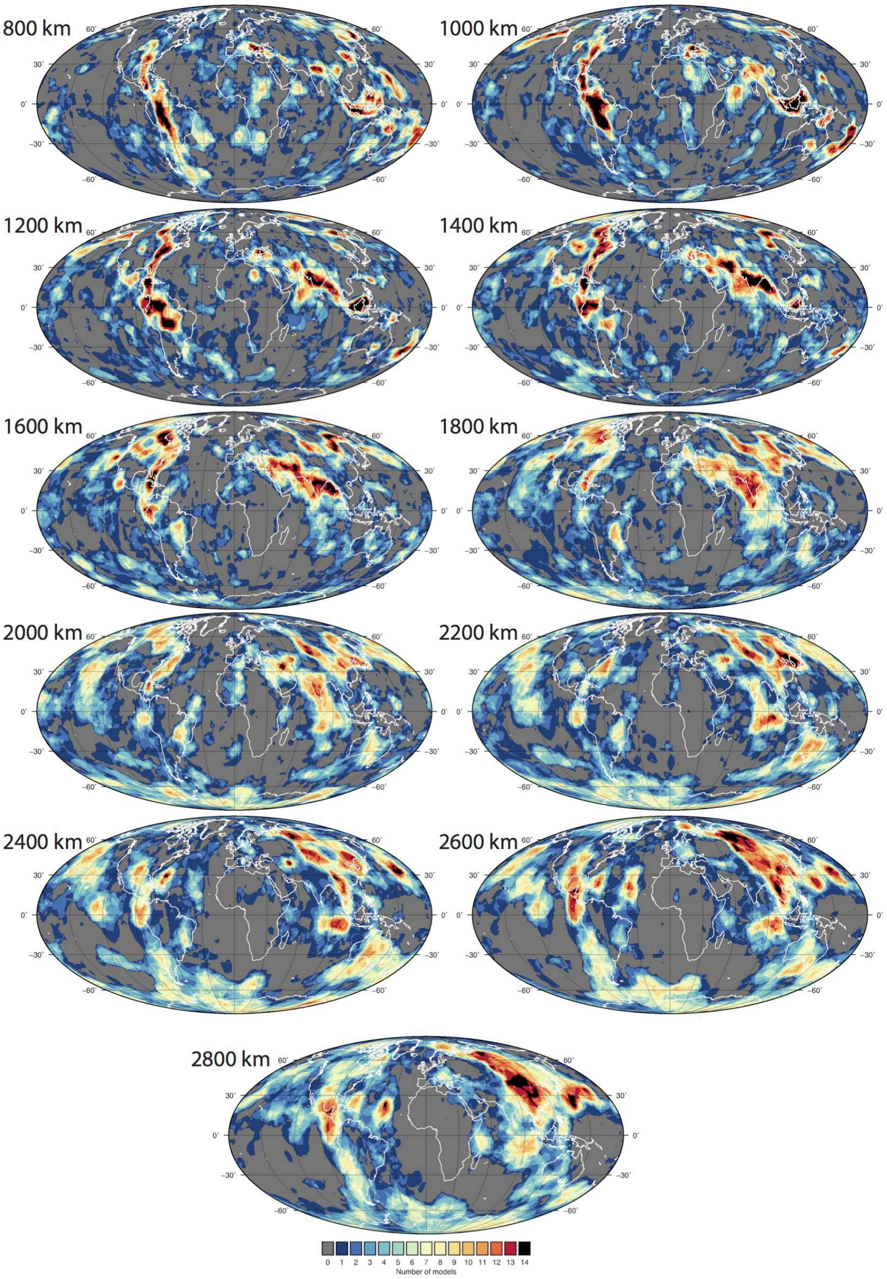

Figure S6. Vote maps from 800-2800 km for the 14 combined models. Grids have the LDM removed and the slab contours are based on the statistical root-mean-square (RMS) instead of the MPV. Figures generated using Generic Mapping Tools as in Figure 10 (GMT v5.3.1; http://gmt.soest.hawaii.edu/).

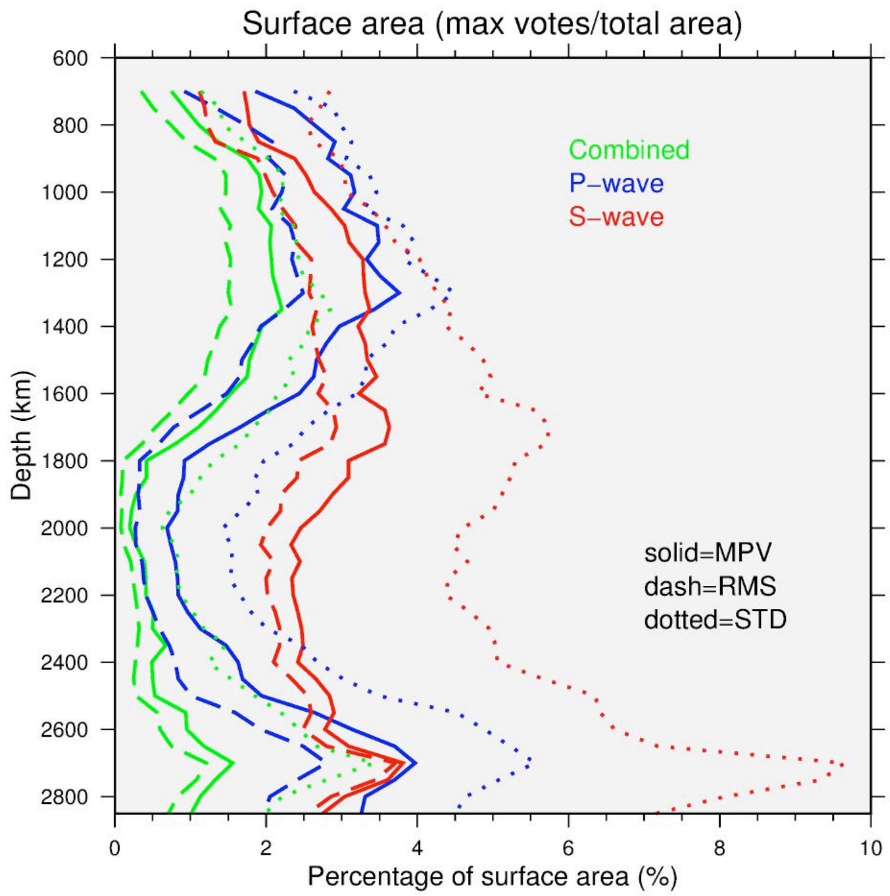

Figure S7. Measure of coverage and agreement of the MPV (LDM removed) contour and vote maps as in Figure 8 compared to RMS (dash) and STD values (dotted).

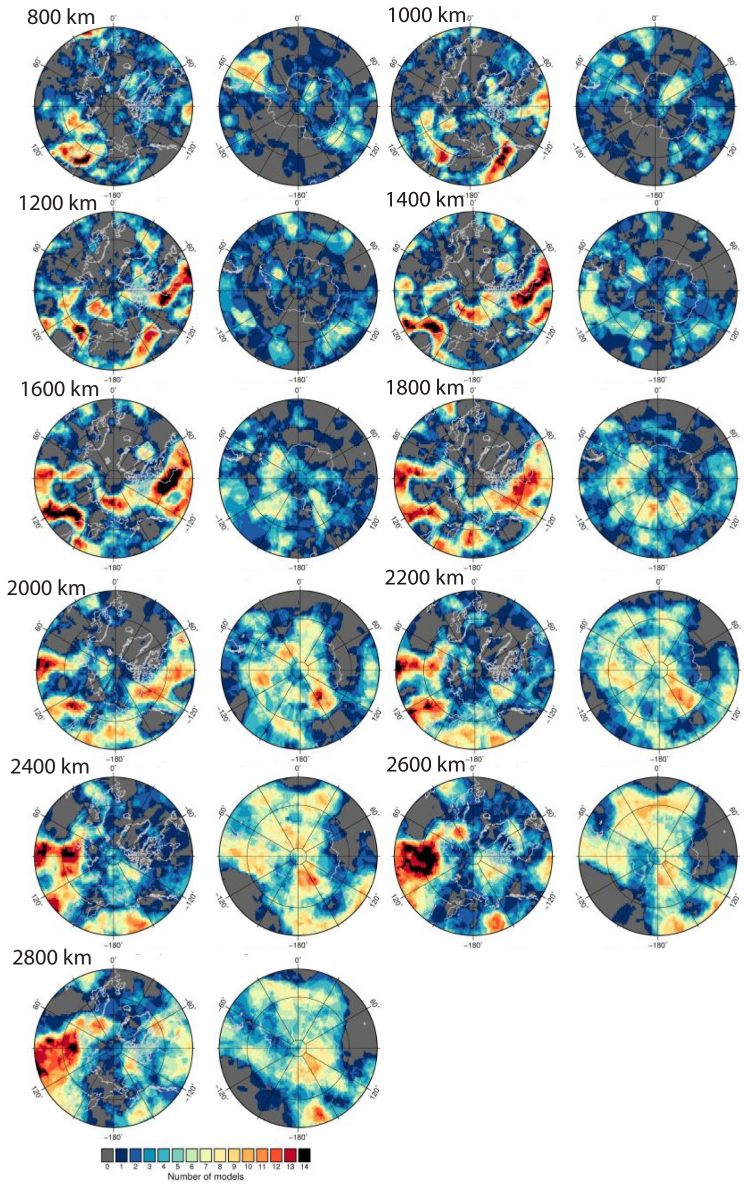

Figure S8. Vote maps from 800-2800 km for the 14 combined models, as in Figure 3 for the reference case, but in a polar projection. Figures generated using Generic Mapping Tools as in Figure 10 (GMT v5.3.1; http://gmt.soest.hawaii.edu/).

Supplementary Table 1. Summary of original depths of the grids for each tomography model.

GAP-P4	GyPSuM-P	HMSL-P06	LLNL_G3Dv3	MITP_2011	PRI-P05	UU-P07	GyPSuM-S	HMSL-S06	PRI-S05	S40RTS	SAVANI	SEMUCB-WM1	TX2011
40	50	66.5	115			5	50	66.5			25		
64.5	137.5	155.5	150			15	137.5	155.5			62.5		
94	212.5	250	185			27	212.5	250			87.5		
129	287.5	350	220			52	287.5	350			112.5		
169	362.5	465	265			90	362.5	465			137.5		
214	462.5	595	310			135	462.5	595			162.5		
264	587.5	735	355			185	587.5	735			190		
319	700	885	410			235	700	885			225		
379	800	1035	411			285	800	1035			272.5		
444	925	1210	450			335	925	1210			335		
514.5	1075	1410	500			385	1075	1410			412.5		
590	1225	1610	550			440	1225	1610			505		
670.5	1375	1810	600			500	1375	1810			610		
756	1525	2010	635			562	1525	2010			730		
846.5	1675	2210	660			627	1675	2210			870		
942	1825	2410	661			650	1825	2410			1020		
1043	1975	2610	721			710	1975	2610			1170		
1149	2125	2798.3501	771			810	2125	2798.3501			1320		
1260	2275		871			920	2275				1470		
1376	2425		971			1040	2425				1620		
1497	2575		1071			1175	2575				1770		
1623.5	2775		1171			1200	2775				1920		
1754.5			1271	Every 50 km	Every 50 km	1325			Every 50 km	Every 25 km	2070	Every 50 km	Every 25 km until 1000 km then every 50 km

1890.5			1371			1500					2220		
2032			1471			1600					2370		
2178.5			1571			1700					2520		
2330			1671			1800					2670		
2486.5			1771			1900					2818		
2723			1871			2000							
			1971			2100							
			2071			2200							
			2171			2300							
			2271			2480							
			2371			2650							
			2471			2815							
			2571										
			2671										
			2741										
			2771										
			2871										
			2891										

Supplementary Table 2. Summary of extracted MPV values for each tomography model for the reference case. Corresponds to Figure 3.

	**GAP-P4**	**GyPSuM-P**	**HMSL-P06**	**LLNL_G3Dv3**	**MITP_2011**	**PRI-P05**	**UU-P07**	**GyPSuM-S**	**HMSL-S06**	**PRI-S05**	**S40RTS**	**SAVANI**	**SEMUCB-WM1**	**TX2011**	**average**	**stdev**	**average P-wave**	**average S-wave**
**700**	0.26	0.25	0.37	0.25	0.17	0.30	0.18	0.47	0.55	0.53	0.46	0.39	0.53	0.43	0.37	0.13	0.25	0.48
**750**	0.26	0.21	0.40	0.25	0.16	0.29	0.16	0.40	0.59	0.52	0.40	0.37	0.48	0.43	0.35	0.13	0.25	0.46
**800**	0.24	0.23	0.35	0.23	0.16	0.28	0.15	0.41	0.51	0.51	0.39	0.35	0.45	0.46	0.34	0.12	0.24	0.44
**850**	0.23	0.20	0.34	0.22	0.16	0.27	0.14	0.34	0.48	0.49	0.38	0.34	0.42	0.46	0.32	0.12	0.22	0.42
**900**	0.21	0.19	0.33	0.22	0.16	0.27	0.13	0.33	0.47	0.48	0.35	0.32	0.41	0.36	0.30	0.11	0.22	0.39

**950**	0.21	0.19	0.29	0.21	0.15	0.26	0.13	0.33	0.42	0.47	0.36	0.31	0.39	0.36	0.29	0.10	0.21	0.38
**1000**	0.19	0.17	0.28	0.20	0.15	0.26	0.13	0.30	0.42	0.45	0.35	0.31	0.39	0.36	0.28	0.10	0.20	0.37
**1050**	0.19	0.16	0.29	0.18	0.15	0.26	0.14	0.31	0.43	0.44	0.35	0.30	0.38	0.33	0.28	0.10	0.20	0.36
**1100**	0.17	0.16	0.26	0.18	0.15	0.25	0.12	0.31	0.38	0.43	0.35	0.30	0.36	0.33	0.27	0.10	0.19	0.35
**1150**	0.18	0.15	0.25	0.19	0.15	0.25	0.12	0.30	0.37	0.42	0.33	0.30	0.34	0.33	0.26	0.09	0.18	0.34
**1200**	0.15	0.16	0.26	0.19	0.15	0.24	0.10	0.31	0.39	0.42	0.32	0.30	0.32	0.32	0.26	0.10	0.18	0.34
**1250**	0.15	0.16	0.25	0.19	0.15	0.24	0.11	0.31	0.36	0.42	0.32	0.30	0.31	0.32	0.26	0.09	0.18	0.34
**1300**	0.14	0.15	0.23	0.18	0.14	0.23	0.12	0.29	0.34	0.41	0.31	0.31	0.30	0.32	0.25	0.09	0.17	0.33
**1350**	0.15	0.14	0.23	0.18	0.14	0.23	0.12	0.30	0.34	0.40	0.30	0.32	0.30	0.32	0.25	0.09	0.17	0.32
**1400**	0.15	0.14	0.26	0.18	0.14	0.22	0.12	0.30	0.36	0.39	0.30	0.32	0.29	0.32	0.25	0.09	0.17	0.33
**1450**	0.15	0.13	0.24	0.16	0.14	0.22	0.12	0.29	0.35	0.39	0.30	0.33	0.30	0.32	0.24	0.09	0.16	0.33
**1500**	0.15	0.13	0.22	0.16	0.14	0.21	0.13	0.30	0.34	0.39	0.29	0.34	0.30	0.31	0.24	0.09	0.16	0.32
**1550**	0.13	0.13	0.22	0.16	0.13	0.21	0.11	0.31	0.36	0.39	0.29	0.34	0.30	0.31	0.24	0.10	0.15	0.33
**1600**	0.13	0.12	0.24	0.15	0.13	0.21	0.09	0.31	0.40	0.39	0.29	0.35	0.29	0.31	0.24	0.11	0.15	0.33
**1650**	0.13	0.13	0.22	0.15	0.12	0.21	0.10	0.33	0.38	0.40	0.30	0.36	0.29	0.33	0.25	0.11	0.15	0.34
**1700**	0.13	0.13	0.21	0.15	0.12	0.21	0.11	0.34	0.37	0.41	0.30	0.36	0.29	0.33	0.25	0.11	0.15	0.34
**1750**	0.13	0.13	0.21	0.15	0.12	0.20	0.10	0.36	0.38	0.41	0.30	0.37	0.29	0.33	0.25	0.11	0.15	0.35
**1800**	0.12	0.14	0.24	0.15	0.12	0.21	0.10	0.40	0.41	0.42	0.30	0.37	0.29	0.36	0.26	0.12	0.15	0.36
**1850**	0.12	0.13	0.22	0.15	0.11	0.21	0.11	0.39	0.39	0.43	0.31	0.38	0.29	0.36	0.26	0.12	0.15	0.36
**1900**	0.13	0.12	0.21	0.14	0.11	0.21	0.12	0.36	0.38	0.43	0.30	0.39	0.30	0.36	0.25	0.12	0.15	0.36
**1950**	0.12	0.11	0.21	0.13	0.11	0.21	0.11	0.37	0.40	0.43	0.31	0.39	0.30	0.36	0.25	0.12	0.14	0.36
**2000**	0.12	0.11	0.23	0.13	0.11	0.21	0.11	0.37	0.43	0.43	0.31	0.39	0.31	0.36	0.26	0.13	0.14	0.37
**2050**	0.12	0.11	0.21	0.14	0.11	0.21	0.11	0.37	0.42	0.43	0.31	0.40	0.31	0.36	0.26	0.13	0.14	0.37
**2100**	0.12	0.11	0.19	0.14	0.11	0.21	0.12	0.39	0.40	0.44	0.31	0.40	0.32	0.37	0.26	0.13	0.14	0.38
**2150**	0.13	0.12	0.20	0.14	0.11	0.20	0.12	0.41	0.41	0.44	0.31	0.41	0.32	0.37	0.26	0.13	0.15	0.38
**2200**	0.13	0.12	0.22	0.13	0.11	0.20	0.13	0.41	0.44	0.44	0.31	0.42	0.32	0.37	0.27	0.13	0.15	0.39
**2250**	0.13	0.13	0.21	0.13	0.11	0.20	0.13	0.43	0.43	0.44	0.31	0.43	0.34	0.42	0.27	0.14	0.15	0.40
**2300**	0.13	0.13	0.20	0.14	0.11	0.20	0.15	0.44	0.42	0.44	0.31	0.44	0.35	0.42	0.28	0.14	0.15	0.40
**2350**	0.14	0.14	0.21	0.16	0.12	0.20	0.13	0.42	0.44	0.45	0.31	0.45	0.36	0.42	0.28	0.14	0.16	0.41
**2400**	0.15	0.15	0.24	0.16	0.12	0.21	0.12	0.43	0.47	0.46	0.31	0.47	0.37	0.43	0.29	0.14	0.16	0.42
**2450**	0.17	0.17	0.24	0.16	0.12	0.21	0.12	0.43	0.47	0.48	0.30	0.49	0.39	0.43	0.30	0.14	0.17	0.43

**2500**	0.18	0.19	0.25	0.18	0.12	0.22	0.12	0.42	0.48	0.50	0.30	0.50	0.41	0.43	0.31	0.14	0.18	0.44
**2550**	0.17	0.22	0.26	0.22	0.13	0.22	0.13	0.44	0.51	0.52	0.32	0.52	0.43	0.49	0.33	0.15	0.19	0.46
**2600**	0.17	0.24	0.28	0.24	0.13	0.23	0.14	0.48	0.55	0.53	0.34	0.54	0.46	0.49	0.34	0.16	0.20	0.48
**2650**	0.17	0.23	0.28	0.24	0.13	0.24	0.16	0.55	0.57	0.55	0.36	0.56	0.49	0.49	0.36	0.17	0.21	0.51
**2700**	0.19	0.24	0.28	0.26	0.13	0.24	0.15	0.63	0.60	0.57	0.39	0.58	0.54	0.81	0.40	0.22	0.21	0.59
**2750**	0.20	0.26	0.30	0.27	0.13	0.25	0.16	0.73	0.65	0.58	0.43	0.60	0.61	0.81	0.43	0.23	0.22	0.63
**2800**	0.20	0.28	0.33	0.27	0.13	0.25	0.17	0.78	0.71	0.59	0.49	0.62	0.71	0.81	0.45	0.25	0.23	0.67
**2850**	0.20	0.28	0.33	0.27	0.13	0.25	0.18	0.78	0.71	0.60	0.56	0.63	0.86	0.81	0.47	0.26	0.23	0.71
**average**	0.16	0.17	0.26	0.18	0.13	0.23	0.13	0.40	0.45	0.46	0.34	0.40	0.38	0.41				
**stdev**	0.04	0.05	0.05	0.04	0.02	0.03	0.02	0.12	0.09	0.06	0.06	0.09	0.12	0.14				

Supplementary Table 3. Summary of % surface area of maximum votes (14 only for the combined case, 7 only for the individual wave cases). Corresponds to Figure 8.

Depth (km)	Combined	P-waves	S-waves	Depth (km)	Combined	P-waves	S-waves
**700**	0.75	1.86	1.71	**1850**	0.42	0.91	3.09
**750**	0.93	2.37	1.75	**1900**	0.28	0.84	2.88
**800**	1.11	2.64	1.78	**1950**	0.22	0.83	2.71
**850**	1.37	2.91	1.9	**2000**	0.2	0.69	2.46
**900**	1.75	2.82	2.38	**2050**	0.28	0.74	2.33
**950**	1.91	3.12	2.53	**2100**	0.39	0.8	2.45
**1000**	1.94	3.17	2.64	**2150**	0.41	0.83	2.34
**1050**	1.9	3.03	2.86	**2200**	0.41	0.84	2.36
**1100**	2.07	3.47	3.04	**2250**	0.51	0.96	2.42
**1150**	2.05	3.49	3.1	**2300**	0.5	1.13	2.47
**1200**	2.07	3.33	3.28	**2350**	0.67	1.47	2.49

**1250**	2.09	3.51	3.29	**2400**	0.49	1.63	2.42
**1300**	2.15	3.76	3.31	**2450**	0.5	1.69	2.66
**1350**	**2.21**	3.43	3.37	**2500**	0.53	1.94	2.84
**1400**	1.95	2.97	3.22	**2550**	0.94	2.64	2.9
**1450**	1.86	2.8	3.31	**2600**	0.95	3.14	2.78
**1500**	1.78	2.67	3.34	**2650**	1.18	3.7	3.09
**1550**	1.75	2.63	3.46	**2700**	1.56	**3.97**	**3.81**
**1600**	1.53	2.44	3.23	**2750**	1.33	3.7	3.61
**1650**	1.34	2.03	3.57	**2800**	1.13	3.31	3.04
**1700**	1.11	1.66	3.63	**2850**	1.01	3.26	2.75
**1750**	0.8	1.25	3.57	**average**	1.15	2.30	2.85
**1800**	0.42	0.92	3.09	**stdev**	0.66	1.06	0.53
